# Obesity results in adipose tissue T cell exhaustion

**DOI:** 10.1172/jci.insight.139793

**Published:** 2021-04-22

**Authors:** Cara E. Porsche, Jennifer B. Delproposto, Lynn Geletka, Robert O’Rourke, Carey N. Lumeng

**Affiliations:** 1 Graduate Program in Immunology,; 2 Department of Pediatrics and Communicable Diseases, and; 3 Department of Surgery, University of Michigan Medical School, Ann Arbor, Michigan, USA.; 4 Department of Surgery, Ann Arbor Veterans Affairs Healthcare System, Ann Arbor, Michigan, USA.

**Keywords:** Inflammation, Metabolism, Adipose tissue, Obesity, T cells

## Abstract

Despite studies implicating adipose tissue T cells (ATT) in the initiation and persistence of adipose tissue inflammation, fundamental gaps in knowledge regarding ATT function impedes progress toward understanding how obesity influences adaptive immunity. We hypothesized that ATT activation and function would have tissue-resident–specific properties and that obesity would potentiate their inflammatory properties. We assessed ATT activation and inflammatory potential within mouse and human stromal vascular fraction (SVF). Surprisingly, murine and human ATTs from obese visceral white adipose tissue exhibited impaired inflammatory characteristics upon stimulation. Both environmental and cell-intrinsic factors are implicated in ATT dysfunction. Soluble factors from obese SVF inhibit ATT activation. Additionally, chronic signaling from macrophage major histocompatibility complex II (MHCII) is necessary for ATT impairment in obese adipose tissue but is independent of increased PD1 expression. To assess intracellular signaling mechanisms responsible for ATT inflammation impairments, single-cell RNA sequencing of ATTs was performed. ATTs in obese adipose tissue exhibit enrichment of genes characteristic of T cell exhaustion and increased expression of coinhibitory receptor *Btla*. In sum, this work suggests that obesity-induced ATTs have functional characteristics and gene expression resembling T cell exhaustion induced by local soluble factors and cell-to-cell interactions in adipose tissue.

## Graphical abstract

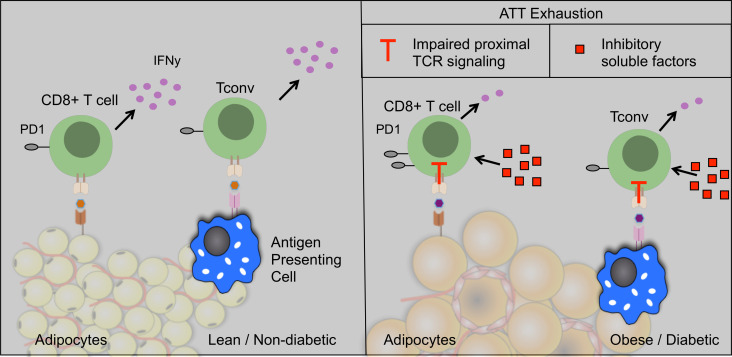

## Introduction

Obesity-associated morbidities such as type II diabetes are characterized by insulin resistance, which is mechanistically linked to adipose tissue dysfunction and inflammation (1–3). Clinical and preclinical studies have identified chronic low-grade inflammation as a critical link between obesity and insulin resistance (4). Immune regulation and systemic metabolism are interconnected within white adipose tissue (WAT). Lean fat has a largely antiinflammatory immune environment containing a predominance of resident adipose tissue macrophages (ATM) and CD4^+^ Tregs (5). However, as lipid storage capacity increases and adipocyte hypertrophy occurs, qualitative and quantitative changes in immune cells also arise. Obese diabetic individuals have a predominance of activated macrophages, proinflammatory CD4^+^ T conventional (Tconv) cells, and CD8^+^ cytotoxic T cells in adipose tissue that correlates with systemic metabolic dysfunction (6–8).

Conventional views of adipose tissue T cell (ATT) functionality indicate that they contribute to the initiation of chronic low-grade inflammation and insulin resistance in obese subjects. CD8^+^ cytotoxic ATTs are required for the development of insulin resistance and infiltrate adipose tissue of high-fat diet–fed (HFD-fed) mice before macrophages (8). Antigen-presenting cells (APC) in obese adipose tissue have also been implicated in activating Tconv with obesity and is dependent on major histocompatibility complex II (MHCII) expression in ATMs, but not adipose tissue DCs (9, 10). Largely, ATTs with antiinflammatory phenotypes decrease (Treg, Th2; refs. 5, 11), and ATTs with proinflammatory phenotypes increase (CD8^+^ and Th1/Tbet^+^ Tconv) in murine and human obese adipose tissue (7, 8, 11). Tregs comprise around 40% of CD4^+^ ATTs in lean tissue, 2–3 times more than is seen in lymphatic tissues (5). Adipose tissue Tregs have tissue-specific characteristics and depend upon the master regulator of lipid metabolism, PPARγ (12). Additionally, adipose Tregs have a distinct clonal T cell receptor (TCR) repertoire, which suggests they are maintained by antigens in lean adipose tissue (13). Tconv also have tissue-specific characteristics. Lean fat contains Tconv with a predominantly tissue resident memory t cell (Trm) phenotype that has been implicated in memory responses to infections (14). With HFD, effector memory adipose tissue Tconv cells with restricted TCR repertoire accumulate, suggesting recognition of antigens specific to obese fat (15). The antigens regulating Tregs and Tconv in lean and obese tissue remain unknown.

While prior work suggests that ATT activation is required for the initiation of adipose tissue inflammation, recent studies call into question the activation state and function of adipose Tconv cells with chronic obesity. Obese adipose tissue contains more PD1^+^ T cells, which have senescent characteristics with impaired IL-2 and IFN-γ production (16). PD1^+^ ATTs secrete osteopontin contributing to inflammation independently of classical T cell activation. Human studies interrogating T cell activation in people with obesity show mixed findings. Obesity has been associated with increased Th1 cells in human adipose and fatty acid metabolites, which activate Th17 T cell inflammation in blood (11, 17). However, studies have also suggested that Th1 T cells from people with obesity are impaired and not hyperactivated. Lymphatic T cells from people with obesity have less potent inflammatory responses that may contribute to impaired responses to viral infections such as COVID-19 (18, 19). Fewer CD69^+^granzyme^+^ cytotoxic T cells and CD69^+^IFN-γ^+^ Th cells are found in obese individuals receiving influenza immunizations compared with lean controls (18). Additionally, preclinical and clinical studies highlight that obesity results in more severe outcomes to influenza infection (20, 21). Whether these T cell impairments result from phenomena such as senescence, anergy, or exhaustion remains unclear. More clarification regarding tissue-specific functionality of ATTs is needed to understand how they contribute to chronic low-grade inflammation and memory responses to infections, and this clarification may provide insight into mechanisms governing global T cell responses in obese patients.

Studies have also been limited by a primary focus on murine WAT, with limited studies assessing functional characteristics of human ATTs. Omental WAT (oWAT) is the prominent visceral fat depot in humans, residing in the peritoneum and covering most abdominal organs (22, 23). In addition to lipid storage, the oWAT plays a substantial role in immunologic surveillance of the peritoneum (24). ATT and B cells are enriched within oWAT and aggregate in structures called fat-associated lymphoid clusters (FALCs; milky spots), which can process and induce an immune response against antigens (25). Understanding how type 2 diabetes mellitus influences human ATT function is of interest for both obesity-induced diabetes and response to foreign peritoneal antigens.

Fundamental gaps in knowledge regarding tissue-specific characteristics of CD4^+^ Tconv and CD8^+^ ATT function impedes progress toward understanding their role in diabetes and adaptive immune responses in diabetic individuals. Many gaps remain in our understanding of how ATT function is shaped by chronic obesity and insulin resistance in humans and mouse models. We do not know tissue-specific characteristics of ATTs in mice or humans, including which cytokines they secrete and their ability to proliferate in response to an activating stimulus. How obesity influences ATT inflammatory potential has not yet been fully elucidated. We sought to answer these questions and hypothesized that HFD feeding would potentiate inflammatory potential in ATTs. However, our findings indicate that obesity results in exhaustion of ATTs and decreased inflammatory potential in obese mice and diabetic humans.

## Results

### ATT activation has tissue-specific characteristics and is dependent upon diet type.

**Figure 1:**
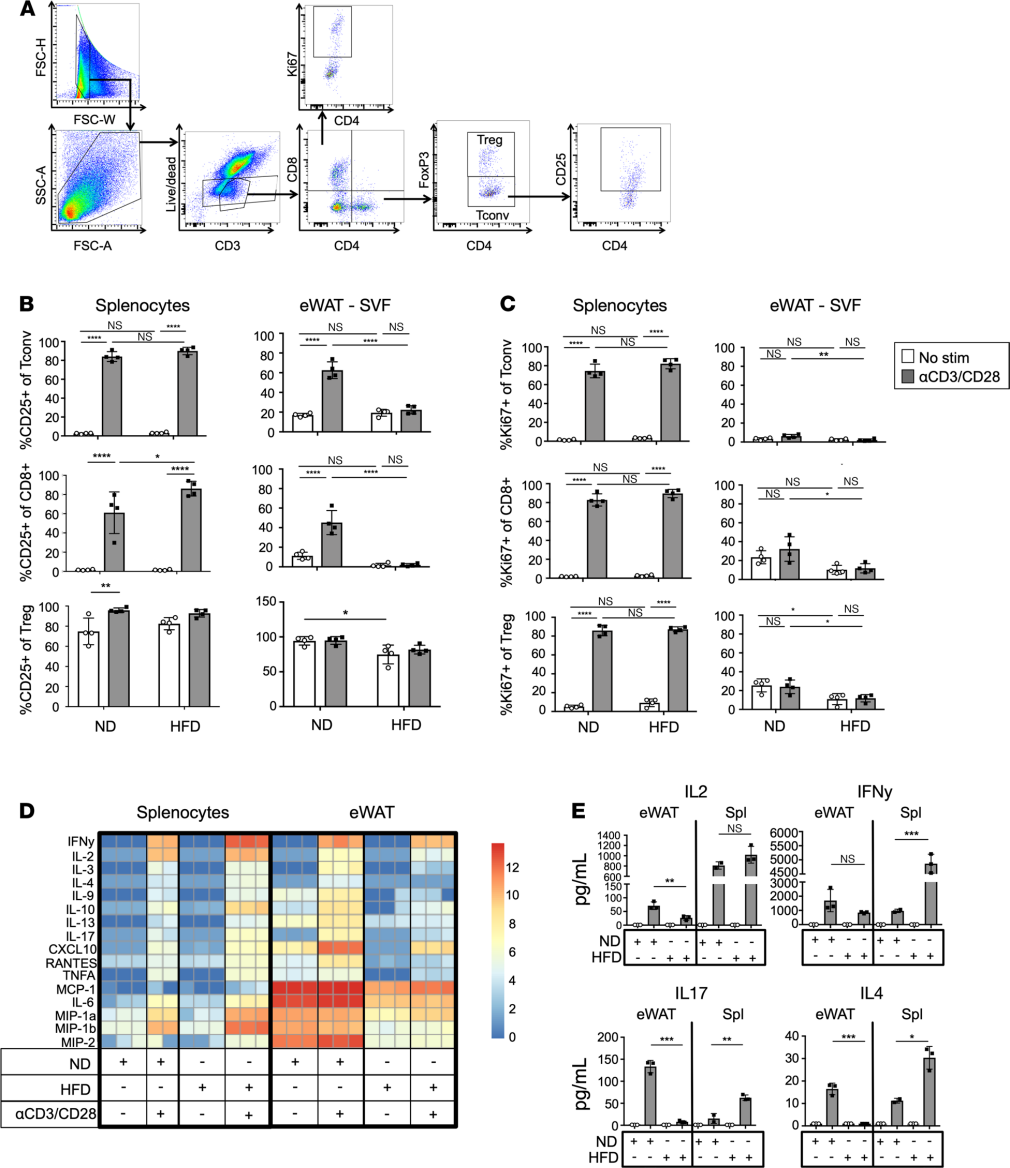
ATT activation capacity is dependent upon diet type. (**A**) Flow cytometry gating strategy used for ATT activation assays. Plot shows eWAT SVF after 3 days of coculture with αCD3/CD28 Dynabeads. Gating is representative of all ATT activation assays. (**B**) Frequency of CD25 expression on Tconv, CD8^+^, and Tregs after T cell activation assays. T cells from splenocytes, oWAT SVF, and eWAT SVF cultures taken from ND- and HFD-fed mice after 18 weeks of feeding. (**C**) Frequency of Ki67 expression on Tconv, CD8^+^, and Tregs from splenocytes and eWAT after T cell activation assays. Cell fractions were assessed with or without αCD3/CD28 Dynabead coculture for 3 days. *n* = 4 biological replicates/group, analyzed by 2-way ANOVA where *P < 0.05, **P < 0.01, ****P < 0.0001. (**D** and **E**) Heatmap of luminex assessment of supernatants taken from T cell activation cultures after 3 days (**D**) and bar graph representation of IL-2, IFN-γ, IL-17, and IL-4 data (**E**) shown in **D**. *n* = 3 biological replicates/group, analyzed by 2-way ANOVA where *P < 0.05, **P < 0.01, ***P < 0.001.

Previous limitations in assessments of ATT function are related to poor viability of ATTs in vitro and the small number of cells that can be collected from a single mouse (26). To overcome these limitations, we devised a culture system where all stromal vascular cells from epididymal WAT (eWAT) were collected and cultured. ATTs were assessed within this heterogeneous fraction. This paradigm allowed for flow analysis of ATTs following in vitro activation and retention of environmental signals from other leukocytes, preadipocytes, and endothelial cells. After 3 days of culture with or without αCD3/CD28 Dynabeads, supernatants were collected and cells were assessed by flow cytometry (Figure 1A). Since Tconv and cytotoxic CD8^+^ T cells upregulate CD25 after receiving stimulus from APC (27), IL-2Rα (CD25) frequency (Figure 1B) and median fluorescence intensity (MFI) (Supplemental Figure 1, A and B; supplemental material available online with this article; https://doi.org/10.1172/jci.insight.139793DS1) were measured on Tconv, CD8^+^ T cells, and Tregs from spleen and eWAT from mice fed normal diet (ND) or HFD for 18 weeks. We noted significant differences in T cell activation depending upon the tissue of origin and diet consumed. Tconv and cytotoxic CD8^+^ T cells from the spleen both increased frequency and MFI of CD25 when stimulated with Dynabeads, as expected. HFD feeding resulted in more robust CD25 expression on splenic CD8^+^ T cells when activated compared with stimulated ND control T cells. However, ATTs had marked differences in their capacity for stimulation based on diet. Unstimulated ATTs had higher basal levels of CD25 expression, and Dynabead stimulation failed to induce CD25 of ND ATTs to the same capacity as those in the splenocyte cultures. Additionally, ATTs from HFD-fed mice are resistant to CD25 upregulation. Unlike Tconv and CD8^+^ T cells, CD25 expression is constitutively expressed on Tregs, as described previously (28, 29). However, CD25 expression was decreased on HFD eWAT Tregs independently of stimulation. Ki67 expression measured by frequency (Figure 1C) and MFI (Supplemental Figure 1, C and D) in Tconv and CD8^+^ T cells was also dependent upon the tissue of origin. In bead-stimulated splenocyte cultures, Ki67 was significantly increased in Tconv and CD8^+^ T cells independent of diet type. However, ATTs were resistant to Ki67 induction in CD8^+^ T cells, Tconv, and Tregs in response to Dynabead stimulus. Total live T cell numbers in cultures also indicate significant differences in the proliferative capacity of splenic and ATT (Supplemental Figure 1, E and F). While splenic T cell numbers significantly increased with TCR stimulus, cell numbers of ATTs present in in vivo cultures remain unchanged with or without Dynabead stimulation.

Cytokine secretion data also indicate impaired HFD ATT inflammatory response to TCR stimulation (Figure 1D). Supernatants from ATT activation assays were collected for assessment by multiplex Luminex assays. T cell effector cytokines were secreted following similar trends as CD25 upregulation. In splenocyte fractions, TCR stimulation significantly increased cytokine secretion, and HFD feeding enhanced effector T cell inflammatory cytokine release of IL-2, IFN-γ, IL-17, and IL-4 (Figure 1E). However, HFD has the opposite effect on ATT inflammatory cytokine secretion. Unlike ND stromal vascular fraction (SVF), which induces a significant increase of Th1, Th2, and Th17 cytokine release with Dynabead stimulation, HFD ATTs fail to induce the same level of cytokine secretion in HFD SVF fractions. ND Rag1-KO SVF was used for ATT activation assays to ensure Dynabead stimulus was not inducing effector T cell cytokines in the absence of ATTs (Supplemental Figure 1G). Overall, these data show that obesity induced by 18 weeks of HFD feeding impairs murine eWAT T cell activation and T cell cytokine production, but it has minimal effects on splenic T cells function.

### ATT activation potential is decreased in diabetic humans.

**Figure 2:**
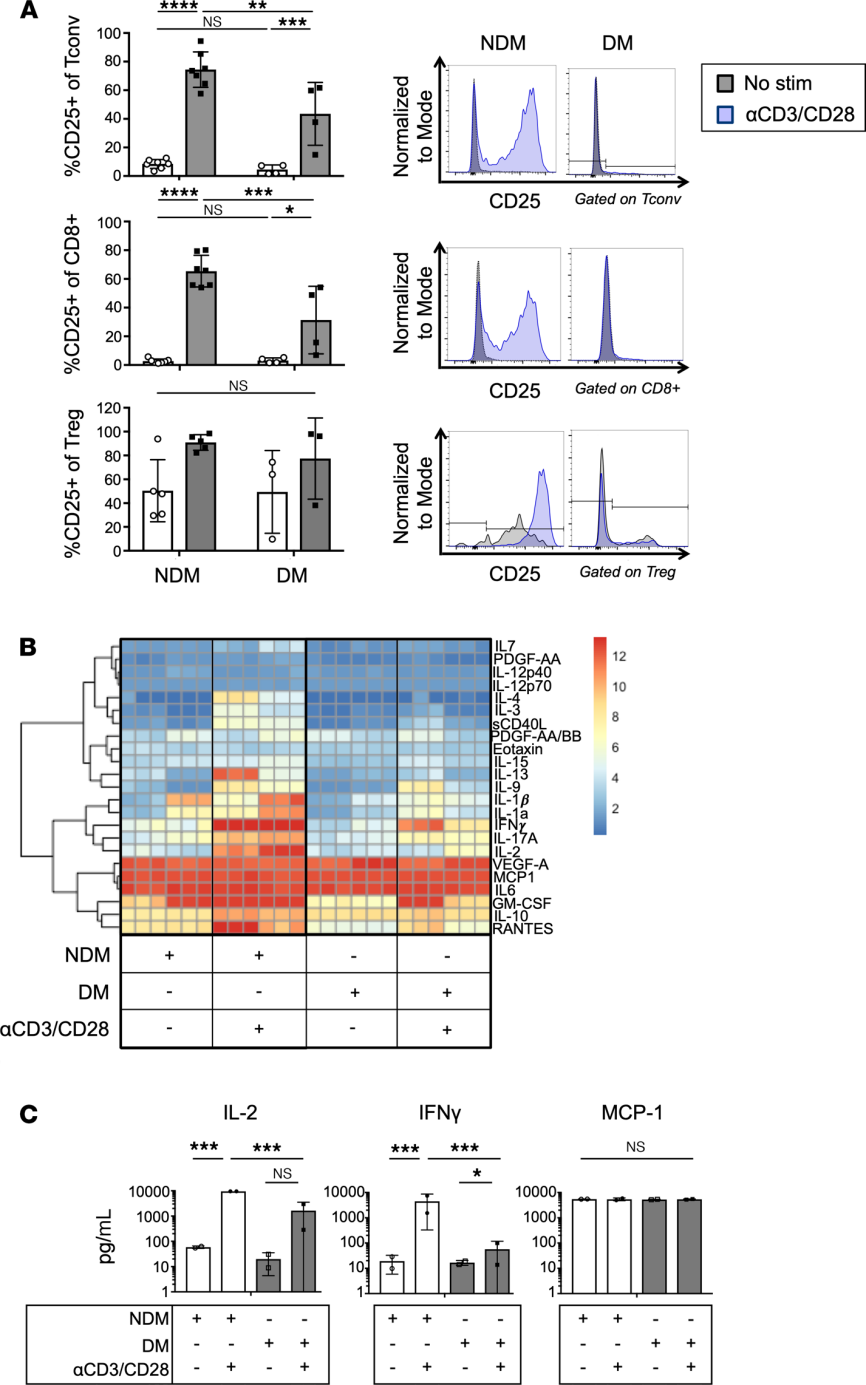
Inflammatory capacity of human ATTs is reduced in diabetic bariatric surgery patients. (**A**) Frequency of CD25 expression on human oWAT ATTs after activation assays with αCD3/CD28 Dynabeads. CD25 induction is compared with the HbA1c of the patient from whom the oWAT biopsy was taken. Representative histograms of CD25 expression compared with unstimulated controls shown on the right. *n* = 4–7 biological replicates/group, analyzed by 2-way ANOVA where *P < 0.05, **P < 0.01, ***P < 0.001, ****P < 0.0001. (**B** and **C**) Luminex assessment of supernatants taken from T cell activation cultures. NDM (HbA1c < 5.7) and DM (HbA1c > 6.5), and IL-2, IFN-γ, and MCP1 cytokines in culture supernatants from human ATT activation assays depicted in bar graphs. *n* = 2 biological replicates/group, 3 technical replicates, analyzed by 2-way ANOVA where *P < 0.05, ***P < 0.001.

Increased Th1 polarized CD4^+^ ATTs have been reported in obese diabetic humans (11). However, our murine culture system indicates that ATTs from obese diabetic tissue have functionally impaired inflammatory properties. Therefore, we assessed human ATTs using omental biopsies from age- and BMI-matched obese male bariatric surgery patients (Table 1). HbA1c levels were used to classify patients as nondiabetic (NDM; < 5.8) or diabetic (DM; > 6.5). ATT activation and inflammatory potential were then measured using the same ATT activation assay used for murine cells. We observed decreased CD25^+^ upregulation in DM ATTs after 3 days of stimulation with αCD3/CD28 Dynabeads (Figure 2A). T cell–specific inflammatory cytokine release was also significantly lower in cells taken from DM patients. Both IL-2 and IFN-γ were significantly increased in culture supernatant from stimulated NDM ATTs, but ATTs from DM human samples were unable to secrete these cytokines to the same degree (Figure 2B). However, MCP1 — a myeloid-derived cytokine — was not significantly different. We performed a Luminex assay to broadly assess effector cytokines from DM versus NDM human SVF cultures (Figure 2C). With ATT simulation, SVF cells from DM humans had a diminished capacity to secrete proinflammatory effector T cell cytokines compared with obese NDM controls. Overall, ATTs from DM visceral human adipose tissue have an impaired inflammatory phenotype upon TCR stimulation, similar to obese diabetic mice.

### ATT dysfunction measured by CD25 expression and cytokine secretion is independently regulated with chronic HFD feeding.

**Figure 3:**
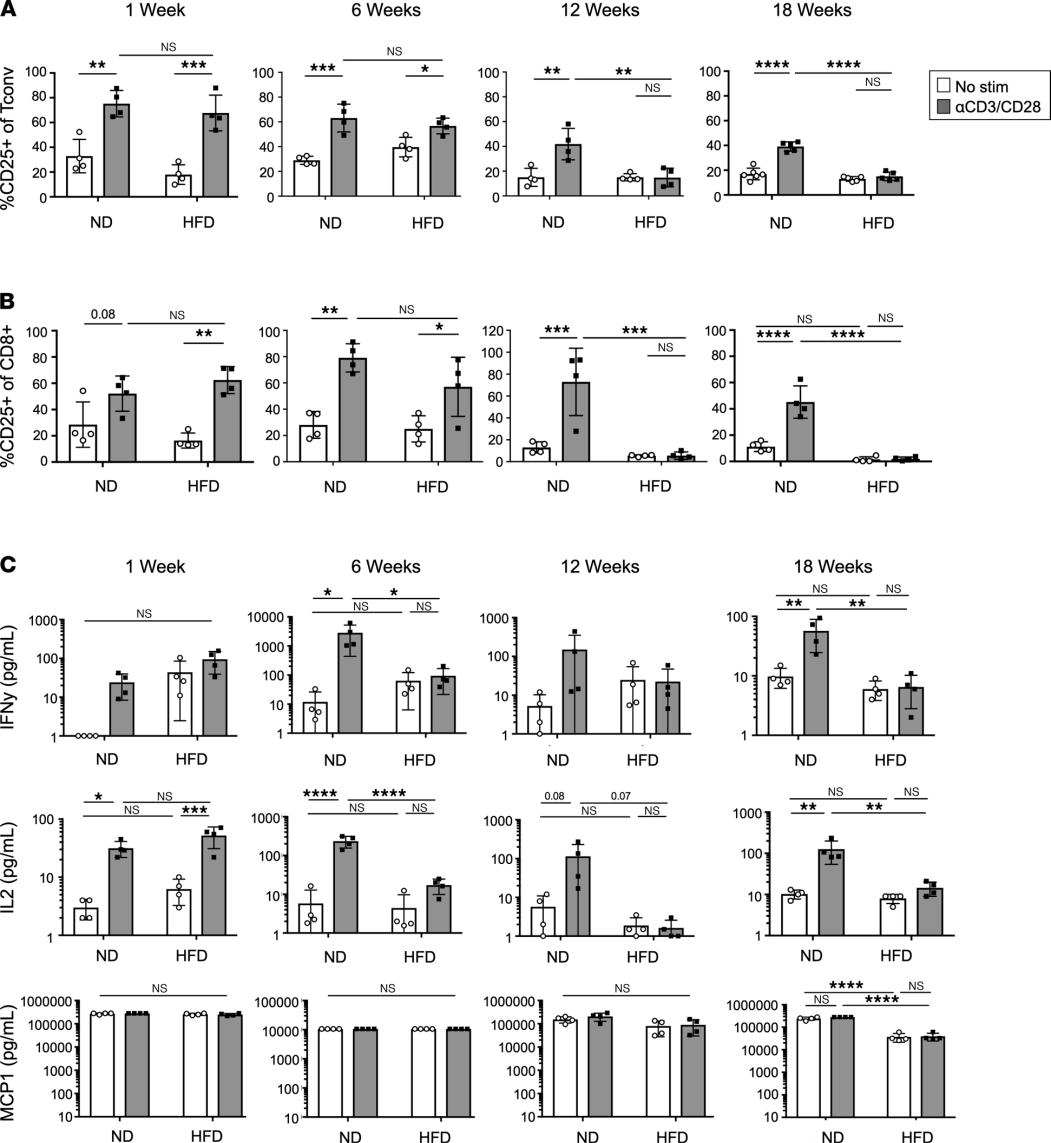
Kinetics of ATT dysfunction. (**A** and **B**) Frequency of CD25 expression on eWAT Tconv and frequency of CD25 expression on eWAT CD8^+^ T cells after ATT activation assays. Assays were performed after 1, 6, 12, and 18 weeks of HFD feeding. (**C**) IL-2, IFN-γ, and MCP1 concentration in supernatants of ATT activation assays at 1, 6, 12, and 18 weeks of HFD feeding. *n* = 4 biological replicates/group, analyzed by 2-way ANOVA where *P < 0.05, **P < 0.01, ***P < 0.001, ****P < 0.0001.

Since diabetic mice and humans have ATTs that are unable to elicit robust inflammatory responses with TCR activation, we examined the kinetics of ATT dysfunction over the course of 18 weeks of HFD feeding. ATT activation assays were used to assess CD25 expression and cytokine secretion from mice fed HFD for 1, 6, 12, and 18 weeks beginning at 6 weeks of age. Following 1 week of HFD feeding, CD25 expression was induced in HFD Tconv (Figure 3A and Supplemental Figure 2A) and CD8^+^ ATT (Figure 3B and Supplemental Figure 2B) to a similar capacity as age-matched ND controls. After 6 weeks of HFD feeding, basal CD25 expression was increased compared with other time points. Although CD25 was increased in ND and HFD ATTs after stimulation, the difference in expression between untreated and TCR-stimulated groups becomes less pronounced. By 12 weeks of HFD feeding, CD25 induction failed to occur after TCR stimulation, and the same trend persisted through 18 weeks. The impairments in ATT cytokine secretion did not correlate with CD25 expression over the time course. IL-2 and IFN-γ were measured as indicators of effector T cell cytokine secretion, and MCP1 was evaluated as a control for innate immune cell function (Figure 3C and Supplemental Figure 2, C, and D). IL-2 and IFN-γ secretion was impaired at 6 weeks of HFD feeding compared with age-matched stimulated controls and persisted through the later time points. MCP1 secretion was not significantly changed based on diet or stimulus, as expected. In sum, ATT dysfunction was not observed with acute HFD feeding and requires an extended period of HFD feeding.

### Macrophage MHCII signals are required for ATT activation impairment.

**Figure 4:**
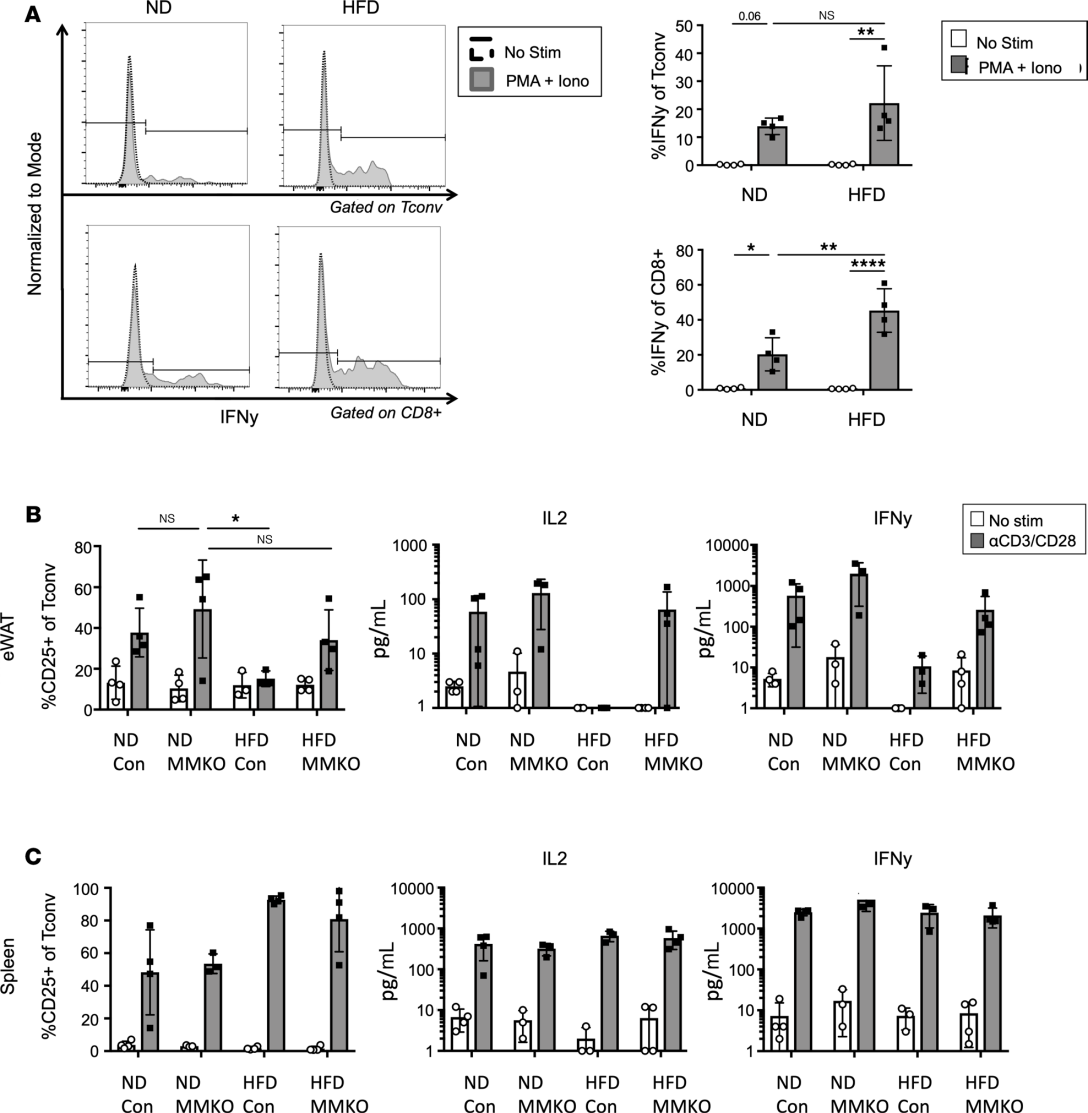
TCR signaling is required for obesity-induced ATT dysfunction. (**A**) Intracellular IFN-γ assessed by flow cytometry in eWAT Tconv and CD8^+^ cells after stimulation with PMA and ionomycin for 4 hours. Representative histograms are shown on the left, and biological replicates are shown on the right. *n* = 4 biological replicates/group, analyzed by 2-way ANOVA where *P < 0.05, **P < 0.01, ****P < 0.0001. (**B**) MMKO mice and age-matched littermate controls were fed ND or HFD for 12 weeks and used for ATT activation assays. eWAT SVF was used for cell surface CD25 expression, and supernatants were used for IL-2 and IFN-γ concentration measurements after 3 days of αCD3/CD28 Dynabead stimulation. (**C**) MMKO splenocytes assessed for cell surface CD25 expression, and supernatants for IL-2 and IFN-γ concentrations after 3 days of αCD3/CD28 Dynabead stimulation. *n* = 3–4 biological replicates/group, analyzed by 2-way ANOVA.

We hypothesized that impairment in TCR signaling pathways may explain decreased ATT activation with chronic HFD feeding. First, Phorbol 12-myristate 13-acetate (PMA)/ionomycin stimulation was used to activate ATTs independently of the TCR. In contrast to αCD3/CD28 stimulation, PMA/ionomycin efficiently induced IFN-γ production in HFD CD8^+^ and Tconv ATTs (Figure 4A). HFD CD8^+^ ATTs had significantly greater IFN-γ production than stimulated ND ATTs. PMA/ionomycin stimulation experiments suggest that HFD ATTs maintain their ability to produce proinflammatory cytokines when activated independently of the TCR. To test whether chronic TCR stimulation is necessary to generate ATT impairment (much like exhausted T cells in tumor microenvironments), we examined ATTs from *MHCII^fl/fl^* × *LysM^Cre^* (MMKO) mice, where MHCII is absent on a majority of macrophages and DCs in adipose tissue (Supplemental Figure 3A) (9). MMKO mice and WT controls were placed on ND or HFD for 12 weeks before analysis of T cell activation (Figure 4B). HFD-fed MMKO mice gained significantly less weight than controls and have decreased proinflammatory ATMs (Supplemental Figure 3, B and C). ATTs from ND MMKO mice were activated to a similar capacity in WT and MMKO cultures based on CD25 expression, IL-2, and IFN-γ secretion. However, HFD-induced impairment of CD25, IL-2, and IFN-γ induction was not observed in MMKO mice. Macrophage MHCII deletion seemed to only impact adipose tissue CD4^+^ ATTs, since splenic T cell inflammatory capacity was unchanged in ND- or HFD-fed mice (Figure 4C), and stimulated CD8^+^ ATTs did not have significant differences in CD25 expression (Supplemental Figure 3D). Overall, these data suggest that ATT impairment in HFD-fed mice required chronic macrophage-derived MHCII signals.

### TCR and coactivating receptors are differentially expressed in obese mice and humans.

**Figure 5:**
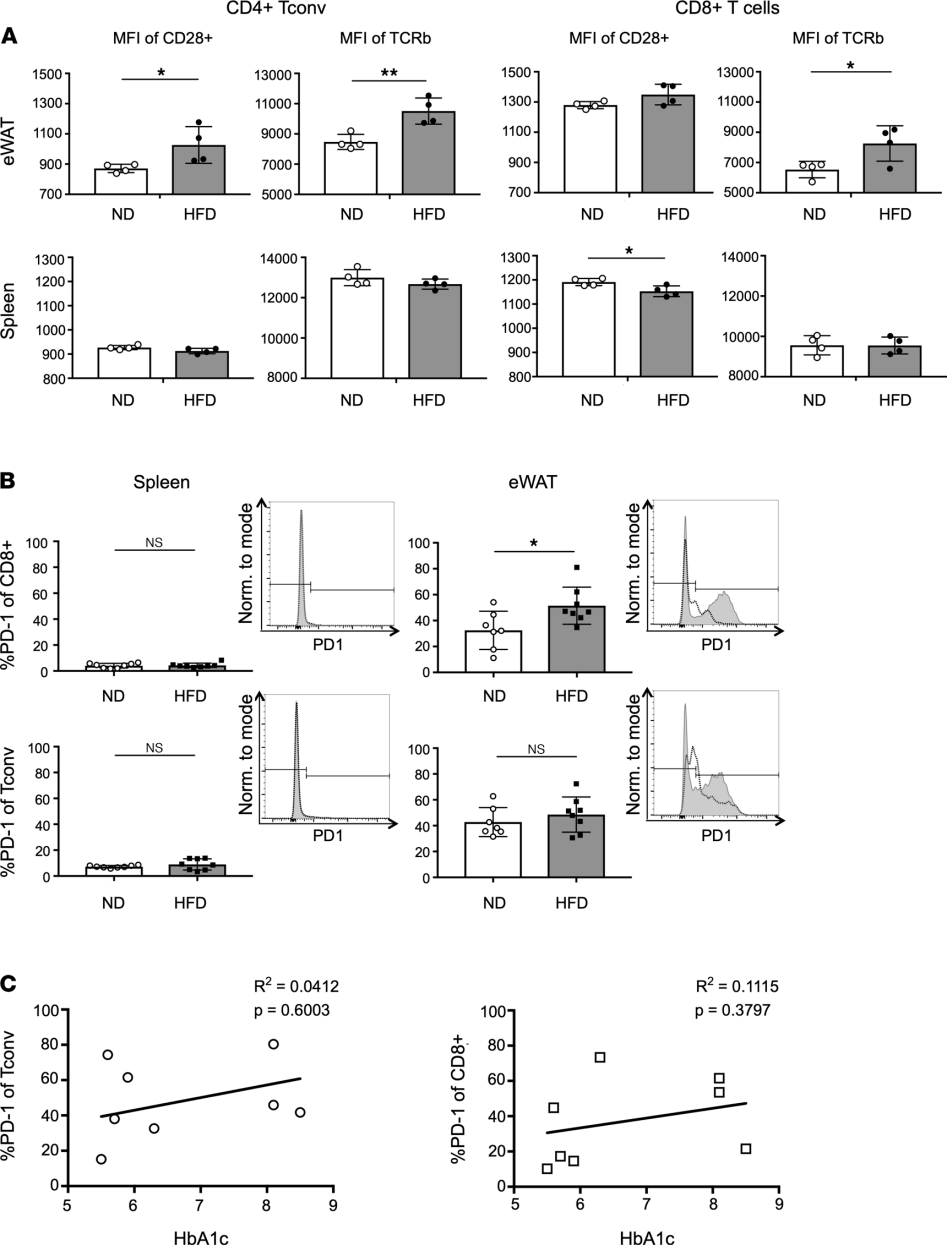
T cell receptor and coreceptor components are differentially expressed in obese mice. (**A**) CD28 and TCR-β expression quantified by MFI of flow cytometry staining on freshly isolated Tconv and CD8^+^ T cells. *n* = 4 biological replicates/group, analyzed by Student’s *t* test where *P < 0.05, **P < 0.01, ***P < 0.001, ****P < 0.0001. (**B**) Frequency of PD1 expression on CD8^+^ T cells and Tconv from freshly isolated eWAT and splenocytes. Representative histograms of PD1 expression are shown on the right of each group. *n* = 7–8 biological replicates/group, analyzed by Student’s *t* test where *P < 0.05. (**C**) Correlation of PD1 expression on Tconv and CD8^+^ ATTs taken from human omentum, compared with patient HbA1c. *n* = 8 biological replicates, using linear regression analysis.

We next assessed whether the lack of TCR and coactivating receptor expression was responsible for their decreased potential for ATT activation. MFI of TCR-β and coactivating receptor CD28 was measured by flow cytometry on freshly extracted eWAT and splenic Tconv and CD8^+^ T cells (Figure 5A). In mice fed HFD for 12 weeks, TCR-β was more highly expressed on Tconv and CD8^+^ cells in eWAT compared with lean mice, and CD28 was more highly expressed in eWAT Tconv. However, TCR-β was not changed by HFD feeding in splenic T cells, and CD28 was significantly decreased on splenic CD8^+^ cells. The expression of TCR coinhibitory receptors — including CTLA-4 on eWAT ATTs (Supplemental Figure 4A) and splenic T cells (Supplemental Figure 4B) — was assessed by flow cytometry. Although eWAT ATTs had higher CTLA-4 expression compared with splenic T cells, expression did not change with HFD feeding. PD1 was more highly expressed on eWAT ATTs than splenic T cells, but the expression on splenic T cells and ATT Tconv cells was not changed by HFD feeding (Figure 5B). However, PD1 was significantly increased in HFD CD8^+^ ATTs. PD1 expression was also assessed on ATTs extracted from human oWAT (Figure 5C). Even though the frequency of PD1-expressing ATTs from human oWAT was substantial, it was not correlated with HbA1c or diabetic status. Overall, these results suggest that loss of TCR and coactivating receptor expression does not correlate with ATT impairment with obesity.

### In vivo PD1 blockade fails to reverse ATT impairment or induce metabolic changes in obese mice.

**Figure 6:**
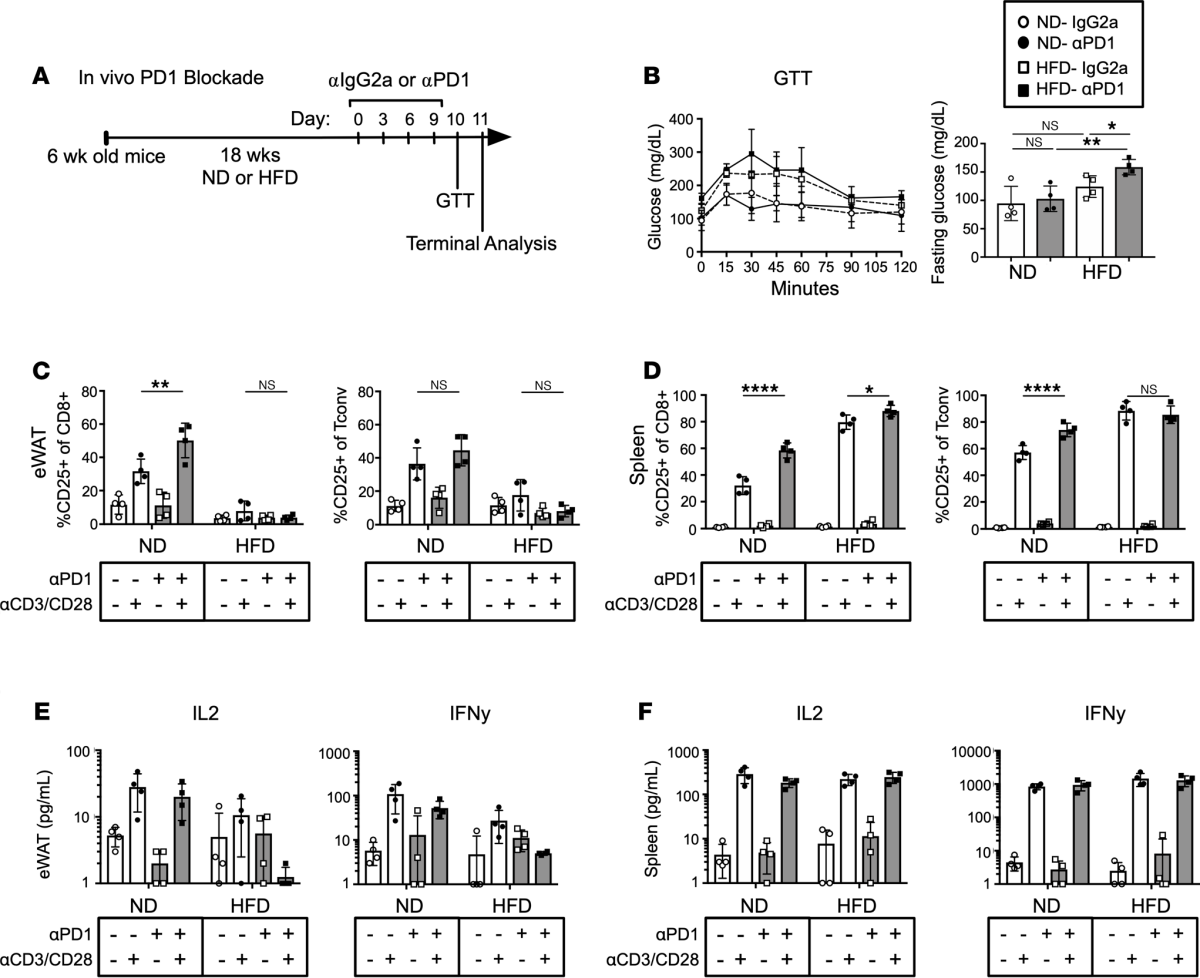
PD1 blockade does not reverse ATT impairment. (**A**) Schematic of in vivo PD1 blockade timeline and interventions. (**B**) Glucose tolerance test of ND- and HFD-fed mice after administration of αPD1 or IgG2a isotype injections (left) and glucose levels after fasting for 6 hours (right). *n* = 4 biological replicates/group, analyzed by 2-way ANOVA where *P < 0.05, **P < 0.01. (**C** and **D**) ATT activation assays were performed on eWAT SVF and splenocytes after PD1 blockade regimen. Frequency of CD25 expression on CD8^+^ T cells and Tconv measured 3 days after Dynabead stimulation. *n* = 4 biological replicates/group, analyzed by 2-way ANOVA where *P < 0.05, **P < 0.01, ****P < 0.0001. (**E** and **F**) IL-2 and IFN-γ concentration in culture supernatants from PD1 blockade ATT activation assays of eWAT SVF and splenocytes. *n* = 4 biological replicates/group, analyzed by 2-way ANOVA.

PD1 is highly expressed in eWAT ATTs, PDL1 is highly expressed on adipocytes (30), and HFD significantly increased PD1 expression in CD8^+^ cytotoxic T cells. Therefore, we performed an in vivo PD1 blockade to evaluate the hypothesis that PD1 expression was required for impaired ATT inflammatory capacity with obesity. Obese mice (18 weeks of HFD) were treated with a PD1 blocking regimen or IgG2a control every third day for 4 total injections, followed by a glucose tolerance test and terminal analysis (Figure 6A). PD1 blockade did not induce a significant difference in body or organ weights (Supplemental Figure 5A). Glucose tolerance was assessed, and obese mice had elevated fasting glucose levels and exhibited glucose intolerance (Figure 6B). However, PD1 blockade did not significantly change glucose tolerance compared with diet-matched IgG2a controls. At the terminal endpoint, eWAT-derived SVF and splenocytes were isolated, and fractions were taken to assess basal T cell composition and binding of αPD1 to ATTs (Supplemental Figure 5B). PD1 antibody injections did not alter frequencies of Tconv, Treg, or CD8^+^ ATTs (Supplemental Figure 5C). However, the PD1 blocking antibody penetrated adipose tissue and was effectively bound to ATTs. ATT activation assays were also performed to assess the inflammatory potential of cells after the PD1 blockade. CD25 expression was increased in stimulated ND eWAT CD8^+^ ATT that received PD1 blockade compared with stimulated isotype control. The same increase in CD25-expressing cells was not seen in ND Tconv populations. More importantly, PD1 blockade did not rescue CD25 expression/upregulation with Dynabead stimulation on HFD CD8^+^ T cells or Tconv cells (Figure 6C). However, PD1 blockade increased CD25 expression on stimulated ND and HFD splenic CD8^+^ T cells, and Tconv from ND mice (Figure 6D). Since CD25 and effector T cell cytokine regulation seems to be regulated independently, we also assessed cytokine secretion in culture supernatants. IL-2 and IFN-γ secretion was not rescued in stimulated ATTs from HFD mice given αPD1. PD1 blockade also did not increase cytokine secretion in activated ATTs from ND mice (Figure 6E). Additionally, PD1 blockade didn’t increase IL-2 or IFN-γ secretion in ND or HFD splenic T cells (Figure 6F). Overall, PD1 blockade can effectively penetrate adipose tissue and bind to PD1-expressing ATTs. However, PD1 blockade did not rescue inflammatory impairment of ATTs in obese mice. It was also unable to prevent the development of ATT impairment in mice given HFD for 6 weeks (Supplemental Figure 6).

### Soluble factors from obese eWAT impair ATT inflammatory capacity.

**Figure 7:**
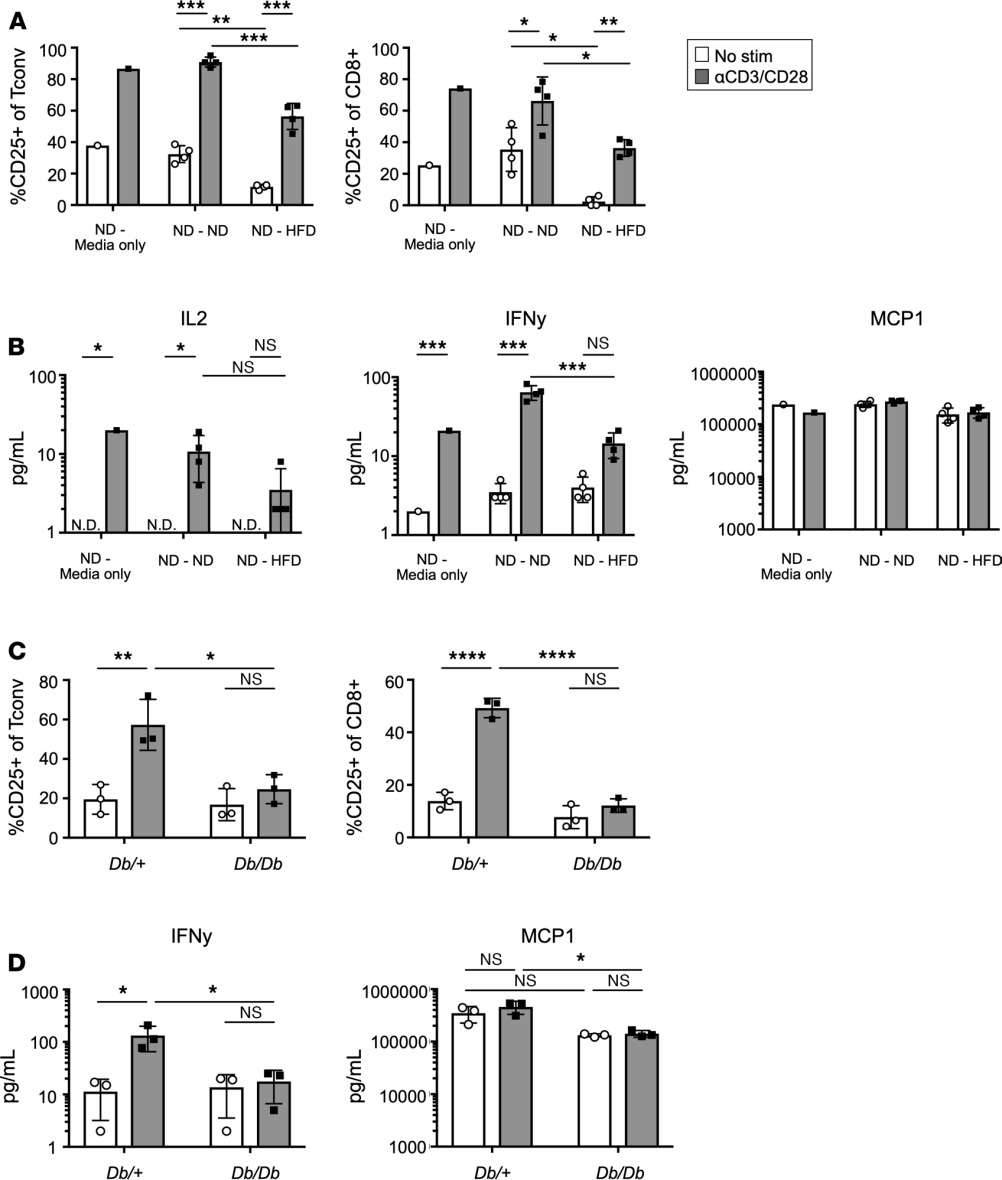
Soluble factors in HFD eWAT partially inhibit inflammatory capacity of ND ATTs. (**A**) Transwell plates were used to coculture ND eWAT SVF (bottom well) with SVF from ND or HFD counterparts in the top chamber. ND SVF in bottom wells were stimulated with αCD3/CD28 Dynabeads and remained cocultured with top chambers for 3 days before analysis. Frequency of CD25 expression on Tconv and CD8^+^ ATT from ND-Media only, ND-ND (ND top chamber), and ND-HFD (HFD top chamber) were compared after ATT activation assay. (**B**) IL-2, IFN-γ, and MCP1 concentration in culture supernatants from coculture ATT activation assays of ND eWAT SVF. *n* = 4 biological replicates/group, analyzed by 2-way ANOVA where *P < 0.05, ***P < 0.001. (**C**) Frequency of CD25 expression on Tconv and CD8^+^ ATTs after ATT activation assay performed on eWAT SVF of 8-week-old *Db/+* and *Db/Db* mice. (**D**) IFN-γ and MCP1 concentrations in supernatants from *Db/Db* ATT activation assay cultures. *n* = 3 biological replicates/group, analyzed by 2-way ANOVA where *P < 0.05.

TCR activation requires both cell-to-cell contact and soluble factors to move T cells from naive to effector states. Testing both seemed to be necessary for a full understanding of potential mechanisms responsible for ATT impairment. To test whether HFD eWAT SVF contains soluble factors that decrease ATT inflammatory potential, transwell coculture studies were employed. ND eWAT SVF was cultured in the bottom wells, while the upper chambers were filled with media only, ND eWAT SVF, or HFD eWAT SVF at 1:1 cell ratio and cocultured for 24 hours before stimulation of the ND SVF with aCD3/CD28 Dynabeads for 3 days. Coculture with ND SVF did not significantly alter CD25 upregulation after stimulation on CD8^+^ T cells or Tconv cells. However, HFD eWAT SVF significantly decreased CD25 expression on both cell types, demonstrating that soluble factors contributed to impairment in T cell activation (Figure 7A). Also, while MCP1 secretion was not impacted by coculture, IL-2 and IFN-γ were decreased when ND eWAT ATTs were cocultured with HFD SVF but not ND SVF (Figure 7B). Since leptin has been reported to contribute to T cell impairment in obesity, we tested if leptin receptor signaling in T cells was required for ATT dysfunction (31). ATTs from leptin receptor–deficient *Db/Db* mice and *Db/+* controls were assessed using the ATT activation assay. CD25 upregulation was not rescued in *Db/Db* mice (Figure 7C). IFN-γ secretion also failed to be restored in obese *Db/Db* mice, while MCP1 was secreted to similar quantities as diet-induced obese mice (Figure 7D). In sum, soluble factors from HFD eWAT SVF contribute to impaired ATT inflammatory potential, and leptin is not a critical mediator.

### Obesity-mediated ATT impairment is associated with T cell exhaustion.

**Figure 8:**
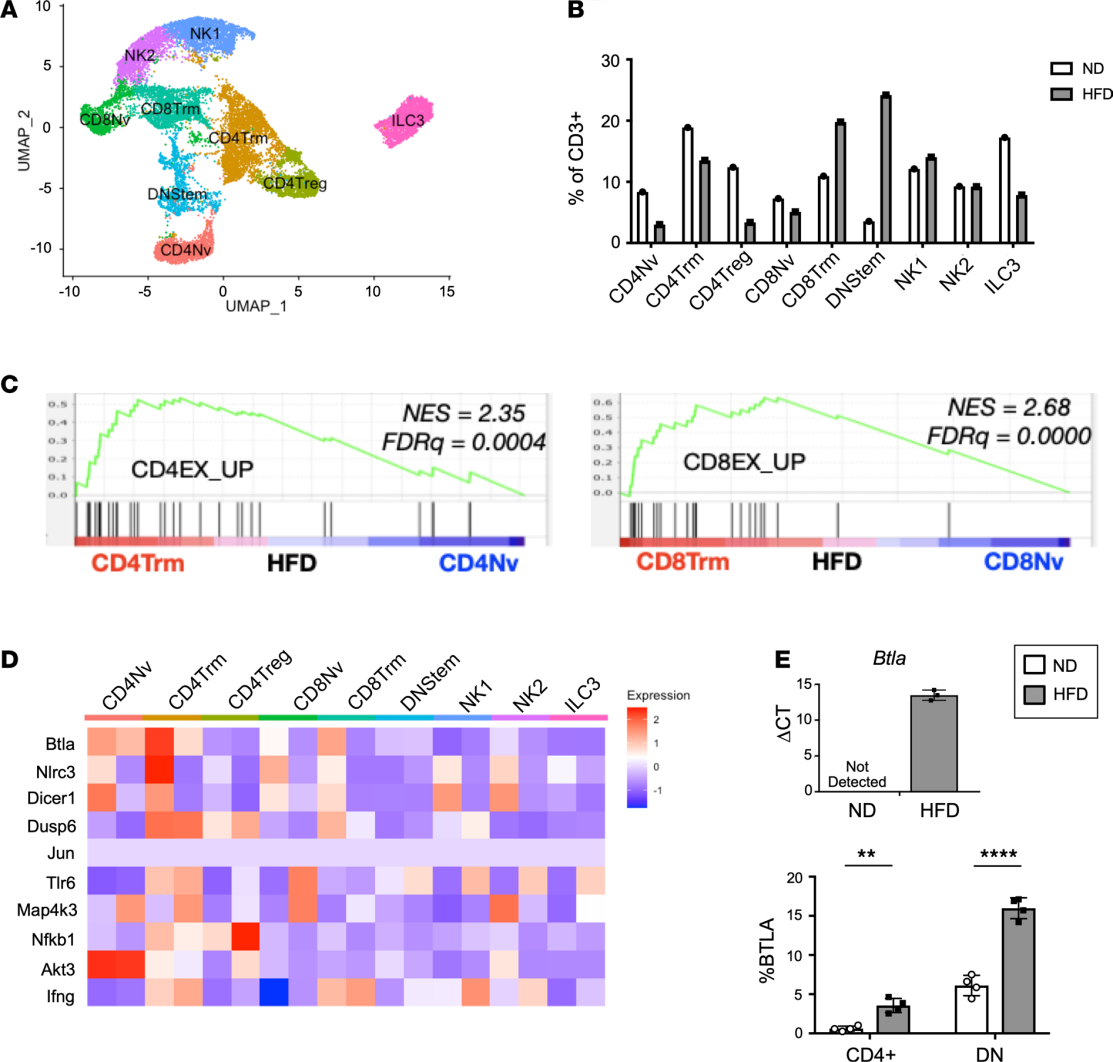
CD4^+^ and CD8^+^ cells from obese adipose tissue exhibit gene enrichment for T cell exhaustion. (**A**) t-SNE plot of CD45^+^CD3^+^ cells sorted from murine eWAT; clustering is based on merged analysis of ND and 12-week HFD-fed samples. (**B**) Frequency of each cluster in ND and HFD of the total CD3^+^ fraction. (**C**) GSEA of HFD CD4Trm versus CD4Nv and HFD CD8Trm versus CD8Nv cells compared against previously published gene expression profiles of exhausted CD4^+^ and CD8^+^ T cells, respectively. (**D**) Heatmap of differentially expressed genes that could be targets for exhaustion in each ATT cluster. (**E**) Quantitative PCR of *Btla* expression from sorted CD3^+^ eWAT ATTs (*n* = 3 biological replicates/group, analyzed by Student’s *t* test), and flow cytometry analysis of BTLA expression on CD4^+^ and DN CD3^+^ ATTs (*n* = 4 biological replicates/group, analyzed by Student’s *t* test where **P < 0.01, ****P < 0.0001).

To complement our ex vivo studies, single-cell RNA sequencing (scRNA-seq) on CD3^+^ cells sorted from murine eWAT was used to assess intracellular signaling pathways governing ATT impairment in obese adipose tissue in vivo. In total, 12,447 cells from ND (*n* = 4 mice pooled) and 4309 cells from HFD (*n* = 2 mice pooled) were collected for scRNA-seq analysis. We identified 9 distinct ATT subtypes within the CD3^+^ ATT fraction (Figure 8A). We defined each subset of CD3^+^ cells based upon the genes that were most enriched in each cluster using cell type–specific genes delineated by literature and Immunological Genome Project (ImmGen) (32). CD4 naive (CD4Nv), CD4 T resident memory (CD4Trm), CD4Treg, CD8Nv, CD8Trm, Double-negative stem (DNStem), NK1, NK2, and type 3 innate lymphoid cells (ILC3) were identified (Supplemental Figure 7). The frequency of these populations was altered by 12 weeks of HFD (Figure 8B). Gene set enrichment analysis (GSEA) against published transcriptional profiles of exhausted and memory T cells were performed. In HFD-fed mice compared with naive ATTs, CD4Trm and CD8Trm had a significant enrichment for genes associated with exhausted CD4^+^ and CD8^+^ T cells, respectively (Figure 8C). None of these pathways were significantly enriched in comparisons of ATTs from ND mice. We identified numerous genes that were differentially expressed in ND and HFD ATTs (Figure 8D). Among the candidate genes was *Btla,* a gene identified as contributing to T cell exhaustion (33, 34). We observed an increase of *Btla* expression in FAC-sorted HFD CD3^+^ ATTs, and expression was not detected in ND CD3^+^ ATTs. Additionally, BTLA expression was assessed by flow cytometry, where increased frequency of BTLA^+^ ATTs was observed in total CD4^+^ ATT and CD4^–^CD8^–^CD3^+^ DN populations (Figure 8E). In sum, obesity induces gene expression that resembles T cell exhaustion in CD4Trm and CD8Trm clusters.

## Discussion

This study has uncovered several potentially novel findings regarding ATT biology in the context of obesity. Previous studies have implicated ATTs as contributors to the initiation of low-grade inflammation in obese adipose tissue contributing to insulin resistance (7, 8, 35). However, more recent studies have found PD1^+^ ATTs in obese tissue have senescent characteristics with decreased classical effector cytokine response after stimulation (16). T cell activation assays have not previously been performed on ATTs, and few studies have interrogated the inflammatory capacity of ATT in vivo. Our in vitro culture assays allowed us to directly assess ATT inflammatory potential in response to TCR activation — a crucial function of T cells to generate adaptive immunity. Although in vitro studies cannot account for tissue architecture, fatty acid availability, and adipocyte signals found in adipose tissue, culturing ATTs within a heterogeneous SVF fraction allowed us to maintain many of the necessary environmental signals present in vivo. We found that HFD-induced obesity dampens the inflammatory capacity of Tconv and CD8^+^ ATTs, which lose their ability to respond to a TCR-specific stimulus. Upon stimulation with αCD3/CD28 Dynabeads, eWAT ATTs from both ND and HFD tissue fails to induce Ki67 expression, unlike splenic T cells. Additionally, ATTs from HFD lose their ability to increase CD25 expression on the cell surface or secrete proinflammatory cytokines, including IFN-γ and IL-2.

We also assessed ATT activation from the human omentum, the major visceral adipose tissue organ. Adipose tissue was collected from a cohort of BMI-matched bariatric surgery patients who were NDM (HbA1c < 5.8) or diabetic (DM, HbA1c > 6.5). Although we hypothesized that obesity and diabetes would result in ATTs with enhanced proinflammatory characteristics, as seen in previous studies by McLaughlin et al. (11), we found that ATTs had decreased inflammatory response to αCD3/CD28 stimulus from metabolically unhealthy patients. Therefore, ATTs from obese adipose tissue in the predominant visceral depot of humans have decreased inflammatory response to TCR-specific stimuli, similar to murine eWAT.

ATT impairment in diabetic human omentum and murine eWAT led us to explore this phenotype further. Since TCR repertoires are restricted on CD8^+^ and Tconv ATTs in obese mice, we hypothesized that ATTs were becoming exhausted due to continuous activation from APC, similar to T cells in tumor microenvironments (15, 36, 37). Considering that a significant percentage of CD4^+^ ATTs are not replenished from circulation but proliferate in situ (14), T cell exhaustion would significantly hinder the antigen-specific and or memory responses. Therefore, we performed a time course comparison of ATT inflammatory potential after Dynabead stimulation. We found that a 1-week short-term HFD failed to inhibit ATT inflammatory potential, indicating that ATT dysfunction is not an acute response to diet. However, after 6 weeks of feeding, ATTs were unable to secrete increased IFN-γ or IL-2 when stimulated with αCD3/CD28 Dynabeads, although they retained their ability to upregulate CD25 expression. This indicates that CD25 expression and cytokine secretion are regulated independently of each other. Based on time point experiments published previously, it also indicates that ATT dysfunction occurs before macrophage infiltration into WAT but after glucose sensitivity starts to change (38, 39). This suggests that, while early obesogenic stimuli trigger ATT activation and promote adipose tissue inflammation, chronic stimulation leads to their functional impairment. This model is consistent with observations of ATT reactivation with weight loss therapies in mice (40, 41). This would imply that ATTs may be dispensable or less important in sustaining adipose tissue inflammation with chronic overnutrition — a concept that is supported by the observations of enhanced adipose tissue inflammation in T cell–deficient mice (42, 43).

Since chronic HFD feeding is required for ATT impairment to occur, we hypothesized that ATTs were becoming functionally exhausted during prolonged periods of exposure to their activating antigen. Bypassing the TCR and activating HFD ATTs with PMA/ionomycin resulted in higher IFN-γ production than ND ATTs, the opposite phenotype of T cell activation driven by αCD3/CD28 TCR stimulus. Previous studies implicating ATTs in chronic low-grade inflammation have assessed ATT phenotypes using PMA/ionomycin stimulation, which explains the previous discrepancies between obesity-driven hyperinflammation of ATTs and senescence (7, 11). Therefore, we tested whether chronic TCR signaling that occurs during HFD feeding was required for decreased response to aCD3/CD28 activation after 12 weeks of HFD feeding. MMKO were used to substantially decrease antigen presentation to ATTs during 12 weeks of HFD feeding. MMKO mice receiving HFD for 12 weeks had ATT inflammatory capacity similar to ND controls when they received an in vitro αCD3/CD28 Dynabead challenge, which did not occur in WT obese controls. This suggested that prolonged signaling through the TCR in WT HFD mice could be required for ATT impairment in obese mice and responsible for T cell exhaustion shown with scRNA-seq. However systemic metabolism could also play an important role in ATT activation capacity, considering that HFD MMKO mice have improved insulin and glucose tolerance compared with HFD WT controls (9). Future studies will need to be performed to assess whether insulin resistance contributes to ATT exhaustion or vice versa.

Since signaling through the TCR is required for ATT impairment, we assessed TCR-β, coactivating, and coinhibitory receptor expression on ATTs from lean and obese mice. TCR-β and CD28 were expressed more highly on ATTs from HFD-fed eWAT than ND controls, indicating that impairment is not due to inadequate TCR expression. Coinhibitory expression is a hallmark of T cell exhaustion, so PD1 and CTLA-4 expression were also assessed. Both proteins were expressed on a larger frequency of ATTs than splenic T cells. However, PD1 blockade did not influence the systemic metabolism of obese mice — as measured by glucose tolerance test (GTT) — and T cell inflammatory potential was not restored, suggesting other mechanisms that may contribute to T cell impairments such as the development of senescence or anergy (44, 45). Although exhaustion, senescence, and anergy all result in impaired T cell inflammatory potential, they have distinct mechanistic characteristics such as expression of E3 ubiquitin ligases, expression of inhibitory receptors, and cell cycle arrest, respectively. To determine what type of inflammatory impairment occurs in obese ATTs, we assessed signaling pathways. We determined that signaling proximally downstream of the TCR is inhibited. Distal signaling pathways seem intact, since PMA/ionomycin induces PKC signaling and calcium influx, and IFN-γ production occurs in HFD ATTs with this stimulation (46, 47).

ND and HFD SVF are exposed to different environmental signals; therefore, we also assessed whether soluble factors in the culture conditions influenced ATT activation, and we determined that soluble factors in the HFD SVF are sufficient to decrease inflammatory potential of ND ATTs. Since leptin receptor signaling in obese mice has previously been implicated in PD1-mediated T cell exhaustion (31), we examined ATTs from *Db/Db* mice and showed that they had an impaired inflammatory phenotype similar to ATTs from diet-induced obese mice, indicating that leptin is not the environmental factor driving ATT dysfunction. Studies using conditional deletion of *Lepr* in CD4^+^ T cells suggests that some splice variants of the leptin receptor may remain active in *Db/Db*, which is a mild limitation of our study (48).

To support our ex vivo observations, we performed scRNA-seq to assess pathways contributing to ATT impairment in vivo after 12 weeks of HFD feeding. CD4Trm and CD8Trm from HFD adipose tissue exhibited characteristics of T cell exhaustion, similar to gene sets related to chronic lymphocytic choriomeningitis virus (LCMV) infection (49). The hallmarks of T cell exhaustion include overexpression of cell surface inhibitory receptors, downregulation of molecules involved in TCR and cytokine receptor signal transduction, and metabolic and bioenergetics deficiencies (50). Specific genes that could be responsible for T cell exhaustion in our data set include B and T Lymphocyte Attenuator *(Btla), Nlrc3*, and *Dicer1* (34, 51, 52). With quantitative PCR (qPCR) and flow cytometry, we show that *Btla* is significantly upregulated in ATTs from HFD mice. This finding is significant because BTLA is a coinhibitory receptor that suppresses TCR signaling in a mechanism distinct from PD1 (34). In humans, BTLA blockade both by itself and in combination with PD1 inhibition has been shown to enhance the inflammatory capacity of T cells (53–55). Therefore, BTLA is a promising target for reversing ATT exhaustion that should be explored further. Additionally, other predicted mechanisms and their influence on TCR signaling should be performed in future studies to elucidate the mechanisms responsible for ATT exhaustion.

Our observations support concepts and mechanisms related to the evidence that obesity and diabetes impair adaptive immune responses. In addition to scRNA-seq data that associate HFD ATTs with T cell exhaustion, we see hallmark characteristics of this impairment, including decreased cytokine secretion and proliferation. These findings are significant because ATTs act as a reservoir of memory cells that can limit reoccurring infections (14). This observation also correlates with inflammatory impairment of lymphatic T cells in obesity. Since lymphatic T cell activation is required to prevent cancer, fight viral infections such as influenza, and induce vaccine immunogenicity (56–58), obesity-induced T cell exhaustion is critical to understand despite its contrast to metabolic chronic inflammation. Therefore, further investigation of ATT exhaustion in people with obesity may be important for resolving T cell–mediated impairments in adipose and nonadipose tissues in patients with obesity.

It is still unknown whether ATT impairment in obese mice and humans is advantageous or inhibits the resolution of chronic low-grade inflammation in adipose tissue. We hypothesize that ATTs become exhausted in the context of obesity as a physiologic feedback mechanism meant to decrease metabolic inflammation that is sustained by macrophages. Decreasing secretion of Th1- and Th17-associated cytokines would reduce the inflammatory tenor of obese individuals. Consistent with this, it has been shown that elevated adipose tissue–associated IL-2 is positively correlated with inflammation and HbA1c (59). However, it is possible that unresolved T cell stimulation through the TCR would result in a lack of inhibitory signals to macrophages, allowing for a continued inflammatory response. Therefore, further studies should be performed to determine the effect of ATT exhaustion in obesity-induced chronic low-grade inflammation.

## Methods

### Animal studies.

C57BL/6J (000664), B6.BKS(D)-Lepr^db^/J (000697), and *MHCII^fl/fl^* (B6.129X1-H2-Ab1^tm1Koni^/J) × *LysM^Cre^* (B6.129P2-Lyz2^tm1cre1fo^/J) mice were obtained from the Jackson Laboratory. MMKO mice were generated by breeding *Lzy2^Cre^* with *MHCII^fl/fl^* mice. Cre-negative littermates were used as controls. Male mice were fed ad libitum either a ND (LabDiet PicoLab, 5L0D; 4.09 kcal/gm, 29.8% protein, 13.4% fat, 56.7% carbohydrate) or a HFD (Research Diets, D12492; 5.24 kcal/gm, 20% protein, 60% fat, 20% carbohydrate) beginning at 6 weeks of age.

### Isolation of murine adipose tissue SVF and flow cytometry analysis.

The SVF was isolated from whole adipose tissue, as previously described (60). Briefly, adipose tissue depots were dissected and weighed. Tissue was then mechanically disrupted by mincing, and it was chemically digested by rocking tissue in 1 mg/mL collagenase IV (Sigma-Aldrich) at 37°C for 30 minutes. Cells were then quenched with RPMI + BSA media and filtered through 100 nm mesh prior to RBC lysis and subsequent filtering with 70 nm mesh filters.

Cells were incubated in Fc Block for 5 minutes on ice before staining with indicated antibodies for 30 minutes at 4°C. Anti-mouse antibodies used included the following: AF488-CD4 (catalog 100423), APCcy7-CD8 (catalog 100713), Brilliant Violet 605-CD279 (PD1) (catalog 135219), PE/Cy7-CD28 (catalog 102125), and APC-TCR-b (catalog 109211) from BioLegend, as well as PerCPcy5.5-CD3 (catalog 45-0031-82), APC-CD25 (catalog 17-0251-82), PE-FoxP3 (catalog 12-4771-82), and PEcy7-Ki67 (catalog 25-5698-82) from eBioscience and Live/dead Fixable Dead Cell Violet Stain Kit (catalog L34955) from Invitrogen.

Stained cells were washed twice with FACS buffer and fixed for intracellular staining using a FoxP3 transcription kit (BD Biosciences). Analysis was performed on an LSR Fortessa Flow Cytometer and analyzed with Flow Jo software (Tree Star Inc.).

### Human samples.

Visceral adipose tissue was collected from male bariatric surgery patients with IRB approval (HUM00074075) from the University of Michigan and Ann Arbor Veterans Affairs Healthcare System. We did not include or exclude any bariatric surgery samples based on race. Sex and racial classifications were made by the participant and extracted from the medical record.

Tissue was finely minced using surgical scissors (DR Instruments) and then digested in 3 mg/mL collagenase II (Invitrogen; 17101015) for 30 minutes. Digested tissue was then processed in the same manner as digested murine adipose tissue to obtain single-cell suspensions of SVF.

Cells were incubated in Fc Block for 5 minutes on ice before staining with indicated antibodies for 30 minutes at 4°C. Anti-human antibodies used included the following: AF488-CD4 (catalog 317419), PerCPy5.5-CD3 (catalog 300429), APC-CD25 (catalog 356109), APCcy7-CD8 (catalog 344713), APC-PD1 (catalog 329907), and PE-FoxP3 (catalog 320107) from BioLegend, and Live/Dead Fixable Dead Cell Violet Stain Kit (catalog L34955) from Invitrogen.

### ATT activation assay.

SVF was extracted from murine eWAT or human oWAT as described in the 2 previous subsections. Splenocytes were also collected and isolated by crushing and rinsing through 70 μm mesh. SVF and splenocytes were treated with RBC lysis buffer (NH4Cl in Tris-HCl buffer) for 5 minutes at room temperature and then quenched with complete RPMI. Cells were counted, and 4 × 10^5^ SVF or splenocytes were plated in 96-well round-bottom plates and rested overnight. The next morning, αCD3/CD28 Dynabeads (Invitrogen) were added to the cultures at a 1:4 bead/cell ratio (as determined by titration experiments). No cytokines or growth factors were added to the media to polarize T cells or stimulate cells otherwise. After 3 days of culture in complete RPMI medium with Dynabeads, 96-well plates were spun down at 450*g* for 7 minutes. Supernatants were aspirated and used for Luminex or ELISA. Cells and Dynabeads were collected in FACS buffer (PBS^–/–^ + 0.5% BSA) and transferred to FACS tubes, which were inserted into EasySep magnets. Cells were transferred into new tubes, while magnetic beads were selectively removed using the magnet. Cells were then washed with 1× PBS before staining with viability dye and extracellular FACS antibodies. Cells were then washed and stained/fixed with intracellular markers as described above.

### PMA/ionomycin T cell activation.

Splenocytes and SVF were purified from spleen and eWAT and were resuspended in a single-cell suspension. Cells were plated at 4 × 10^5^ cells/well in a 96-well round-bottom plate and rested overnight. The next morning, 10 μg/mL of Brefeldin A was added to cultures to block protein secretion from the Golgi apparatus. After 1 hour, SVF was stimulated with 10 ng/mL of PMA and 250 ng/mL of ionomycin. After stimulation for 4 hours, cells were collected, washed, and stained for flow cytometry to assess live ATT subpopulations and intracellular IFN-γ (Alexa Fluor 647 IFN-γ [catalog 505814] from BioLegend).

### Cytokine analysis.

Luminex assays were performed on supernatants collected after 3 days of culture. Murine samples were analyzed with EMD Millipore’s Milliplex MCYTOMAG-70k kit. Human samples were analyzed using EMD Millipore’s Milliplex HCYTOMAG-60k kit. Subsequent cytokine secretion assays were performed on IL-2, IFN-γ, and MCP1 using ELISAs performed in the University of Michigan’s Cancer Center Immunology Core.

### In vivo PD1 blockade.

At 6 weeks of age, C57BL/6J mice were fed ND or HFD for 18 weeks to induce ATT impairment. At 24 weeks of age, mice were given injections of anti–mouse PD1 (catalog CD279, clone RMP1-14, Bio X Cell) or IgG2a isotype control, anti-trinitrophenol (clone 2A3, Bio X Cell) at 10 mg/kg every third day for 4 total injections. The day after the last injection, systemic metabolism was assessed by GTT. After refeeding and resting overnight, mice were sacrificed, and a fraction of fresh SVF was stained for flow cytometry analysis to assess basal T cell differences and ensure the PD1 antibody entered adipose tissue and blocked PD1. The following antibodies were used: AF488-CD4 (catalog 100423) and APCcy7-CD8 (catalog 100713) from BioLegend and PerCPcy5.5-CD3 (catalog 45-0031-82), PE-FoxP3 (catalog 12-4771-82), and Alex Fluor 647 anti–rat IgG2a (catalog 407511) from eBioscience. The remaining SVF was used for an ATT activation assay to assess whether PD1 blockade restored ATT inflammatory potential.

### Metabolic evaluations.

GTT were performed after a 6-hour fast. Mice were injected i.p. with D-glucose (0.7 g/kg), and blood glucose concentrations (mg/dL) were measured at 0, 15, 30, 45, 60, 90, and 120 minutes after injection from tail nick with a glucometer.

### Transwell ATT activation assays for soluble factor analysis.

ND SVF was isolated and plated in the bottom of transwell plates. Ninety-six–well 0.4 μm pore transwell chambers were seeded with ND or HFD SVF at a 1:1 ratio with cells in the bottom wells. Bottom wells and top chambers were cultured separately overnight at 37°C. The next day, upper chambers were cocultured with ND SVF for 24 hours prior to αCD3/CD28 Dynabead stimulation of ND bottom wells. Cells were stimulated and kept in coculture for 3 days, at which point supernatants and cells were taken for ATT activation analysis.

### scRNA-seq.

The SVF was isolated from whole adipose tissue as previously described (Isolation of Murine Adipose Tissue SVF and Flow Cytometry Analysis). CD3^+^ cells were sorted from 3 eWAT of lean and obese mice in duplicate. ATTs were stained for FACS using a Sony MA900 (see Table 2 for antibody details).

Samples were taken to the University of Michigan Advanced genomics core for library construction with the 10× Genomics platform (V2.0) and sequencing (Next-Seq). Count matrices were generated with Cellranger (10× Genomics), with postprocessing using Seurat (v3) (61). Ribosomal and mitochondrial genes were removed prior to analysis, and cells were filtered to remove cells, with < 200 unique genes identified. Following transformation of the data, an integrated analysis of all the depots and conditions was performed. ATT clusters were defined by identifying genes significantly enriched in each cluster compared with the other cells, independent of diet and depot, and comparing them with gene profiles in ImmGen (32). Differentially expressed genes (DEGs) were identified using Wilcoxon rank sum tests adjusting for FDR. DEGs were defined as adjusted *P* value of < 0.05 and log_2_ fold changes > 0.3. Gene pathway enrichment was analyzed using the biological pathways PATHER gene ontology (GO) database. Data sets were then separated by depot of origin, and DEGs for ND and HFD ATTs were assessed for each cluster and each depot. Additional packages used for data analysis to try to remove contaminants include Cellbender, Liger, and SoupX (62–64). This scRNA-seq data set has been deposited in GEO as Series GSE167212 (http://www.ncbi.nlm.nih.gov/geo/query/acc.cgi?acc=GSE167212).

### Btla analysis.

The SVF was isolated from whole eWAT, as previously described (Isolation of Murine Adipose Tissue SVF and Flow Cytometry Analysis). CD3^+^ cells were sorted from 4 lean and obese mice. ATTs were stained for FACS analysis and sorting using a Sony MA900. Btla, CD4, and CD8 antibodies were added into the sorted cells so *Btla* expression could be analyzed on subpopulations of CD3^+^ ATTs. Sorted CD3^+^ cells were taken for RNA extraction using a Qiagen RNA micro kit. cDNA was generated from RNA using high-capacity cDNA reverse transcription kits (Applied Biosystems). Power SYBR Green PCR Master Mix (Applied Biosystems) and the StepOnePlus System (Applied Biosystems) were used for qPCR.

### Statistics.

All values are reported as mean ± SD. Differences between groups were determined using unpaired, 2-tailed Student’s *t* test or 2-way ANOVA with Tukey post hoc tests using Graph Pad Prism 7 software. *P* values less than 0.05 were considered significant.

### Study approval.

All mouse procedures were approved by the University Committee on Use and Care of Animals at the University of Michigan and were conducted in compliance with the *Guide for the Care and Use of Laboratory Animals* (National Academies Press, 2011). All human studies were performed with tissue collected from bariatric surgery patients with IRB approval (HUM00074075) from the University of Michigan and Ann Arbor Veterans Affairs Healthcare System.

## Author contributions

CEP, RO, and CNL designed research and experiments. CEP, JBD, and LG performed experiments. CEP analyzed experiments. CEP and CNL wrote the manuscript. All authors have read and approved the manuscript.

## Supplementary Material

Supplemental data

## Figures and Tables

**Table 2:**
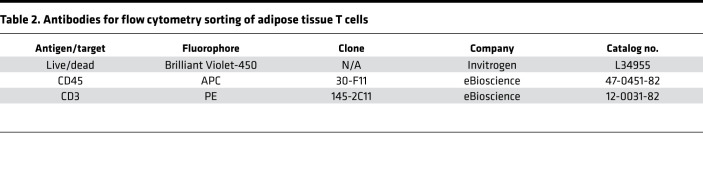
Antibodies for flow cytometry sorting of adipose tissue T cells

**Table 1:**
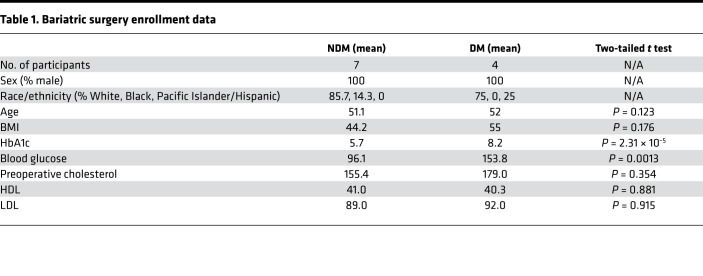
Bariatric surgery enrollment data

## Version 1 03/16/2021

In-Press Preview

## Version 2 04/22/2021

Electronic publication
