# Supplementary material for: Obesity results in adipose tissue T cell exhaustion
Source: JCI Insight. 2021 Apr 22;6(8):e139793. doi: 10.1172/jci.insight.139793 (PMC8119198; doi:10.1172/jci.insight.139793)
Supplement: Supplemental data [file jciinsight-6-139793-s011.pdf]

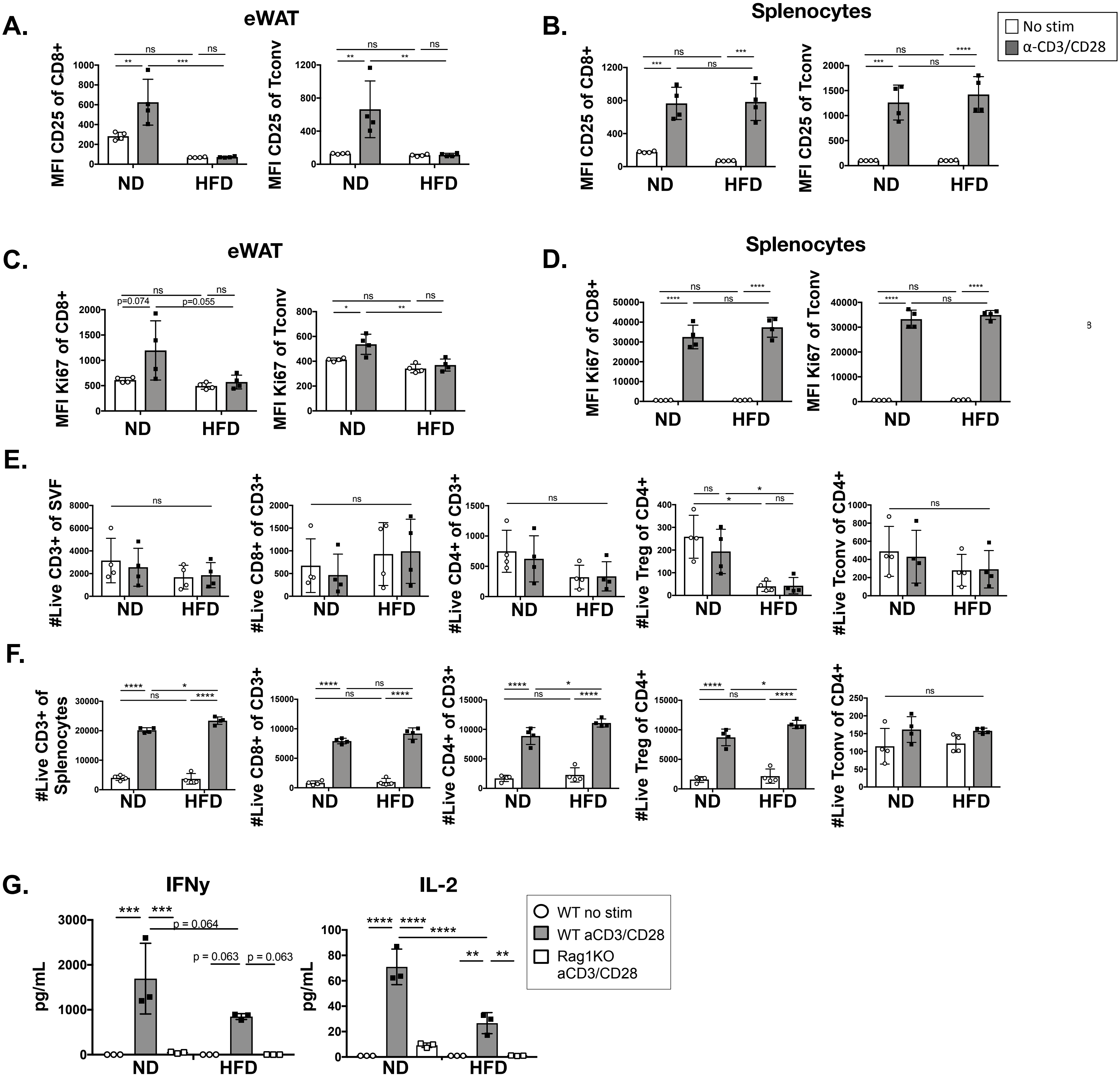

Supplemental Figure 1: Additional descriptive data for ATT activation assay.

(**A**) MFI of CD25, (**B**) MFI of Ki67 in CD8+ and Tconv ATT cells from eWAT after ATT Activation assays. (**C**) MFI of CD25, (**D**) MFI of Ki67 in CD8+ and Tconv ATT cells from Splenocytes after ATT Activation assays. (**E**) Number of eWAT cells, (**F**) Number of splenocytes seen in Live CD3+, total CD4+, Tconv, Treg, and CD8+ subpopulations in ATT activation assays.(**G**) Frequency of live cells in ATT activation cultures from eWAT and splenocytes, (**H**) IFNy and IL2 secretion from WT v Rag1KO SVF with αCD3/CD28 dynabead treatment. (**I**) Bar graphs depicting heat map data in Figure 1E.

* = $p < 0.05$, ** = $p < 0.01$, *** = $p < 0.001$, **** = $p < 0.0001$

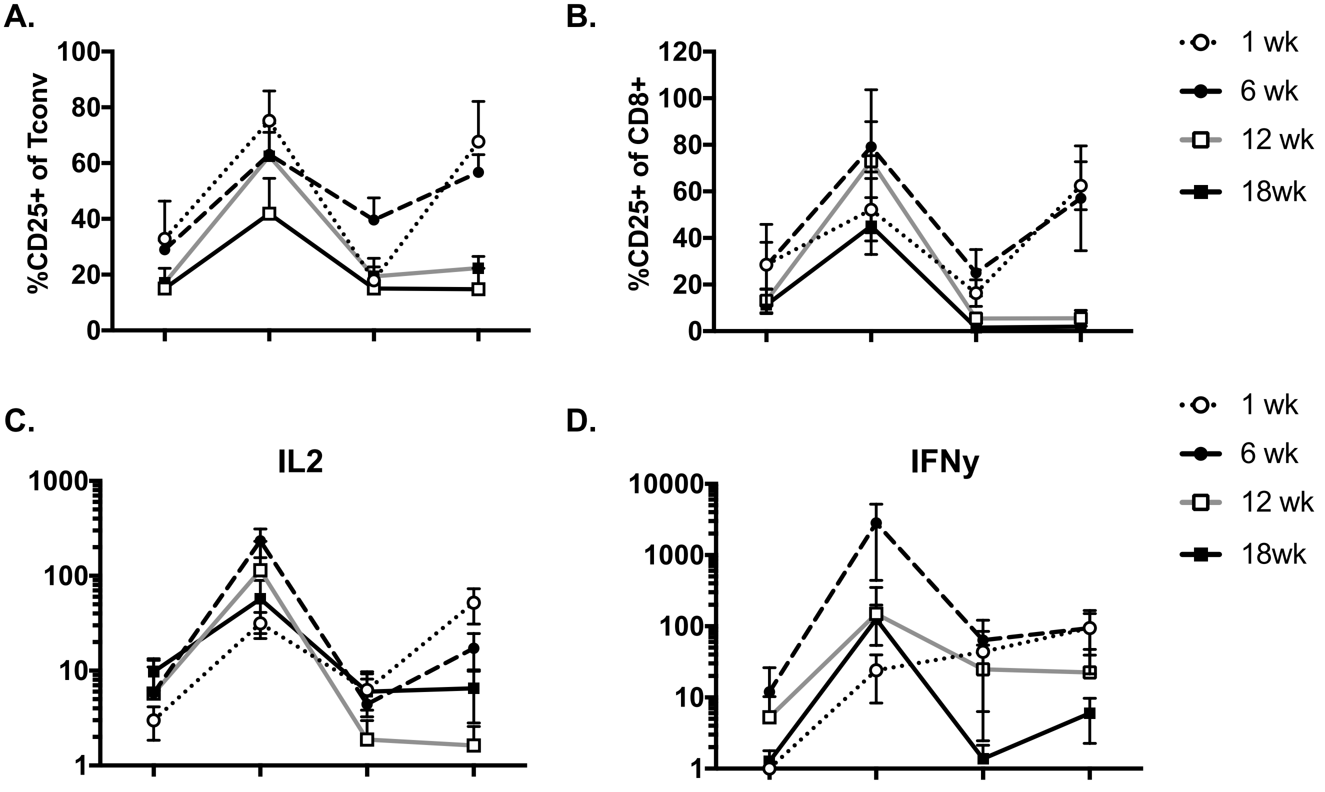

Supplemental Figure 2: Additional descriptive data for ATT activation kinetics.

(**A**) Line graphs of eWAT Tconv and (**B**) eWAT CD8+ ATT activation assays at 1, 6, 12, and 18 weeks of HFD feeding.

* = $p < 0.05$, ** = $p < 0.01$, *** = $p < 0.001$, **** = $p < 0.0001$

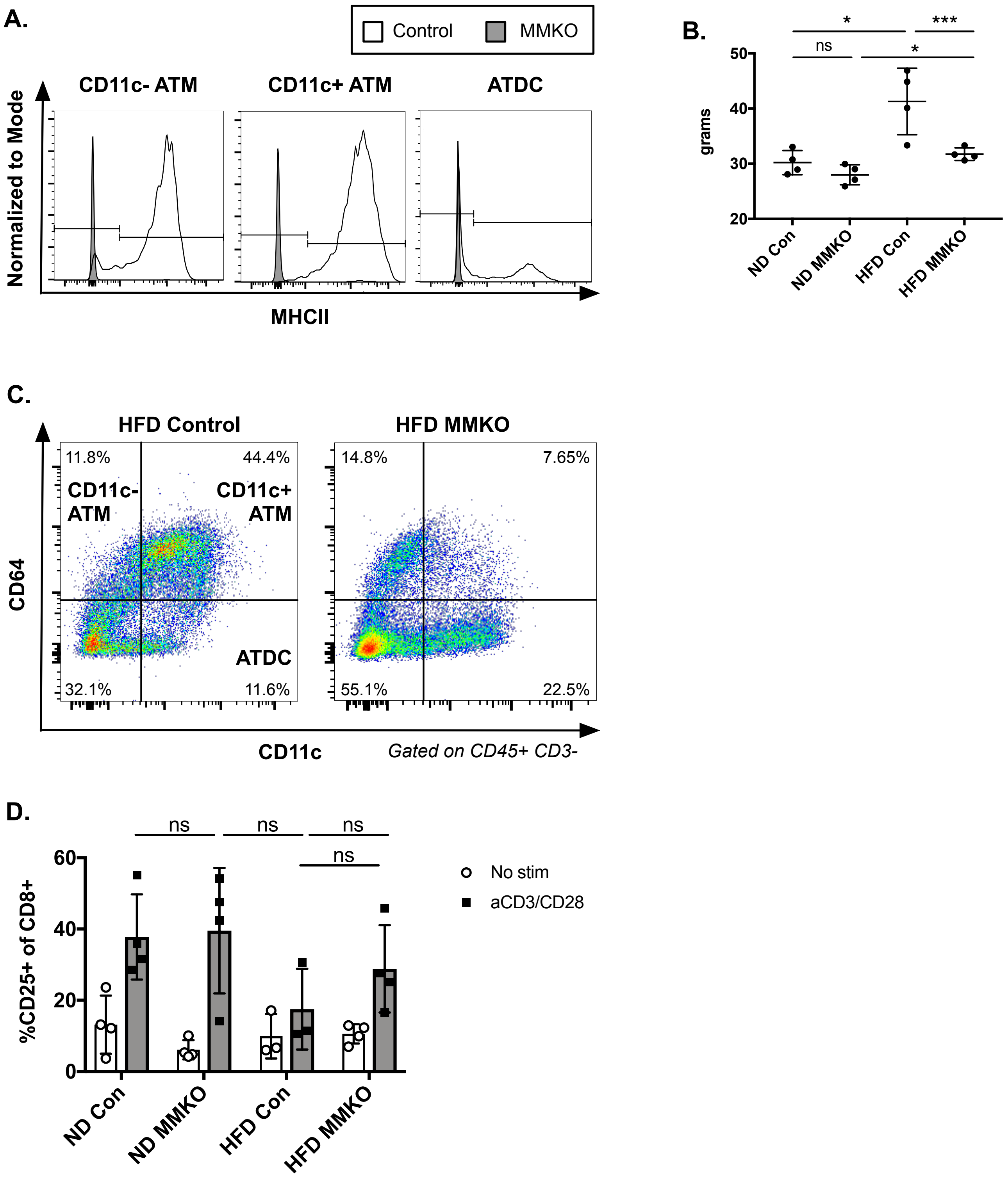

Supplemental Figure 3: Metabolic and immunologic phenotype of HFD MMKO mice.

(**A**) Flow plots illustrating MHCII knockout in the primary antigen presenting cells in HFD adipose tissue.

(**B**) Body weights of MMKO mice and controls.

(**C**) Flow plots illustrating antigen presenting cell subsets in HFD MMKO adipose tissue after 12 weeks.

(**D**) CD25 expression on MMKO CD8+ ATTs from eWAT following ATT activation assays.

* = $p < 0.05$, ** = $p < 0.01$, *** = $p < 0.001$, **** = $p < 0.0001$

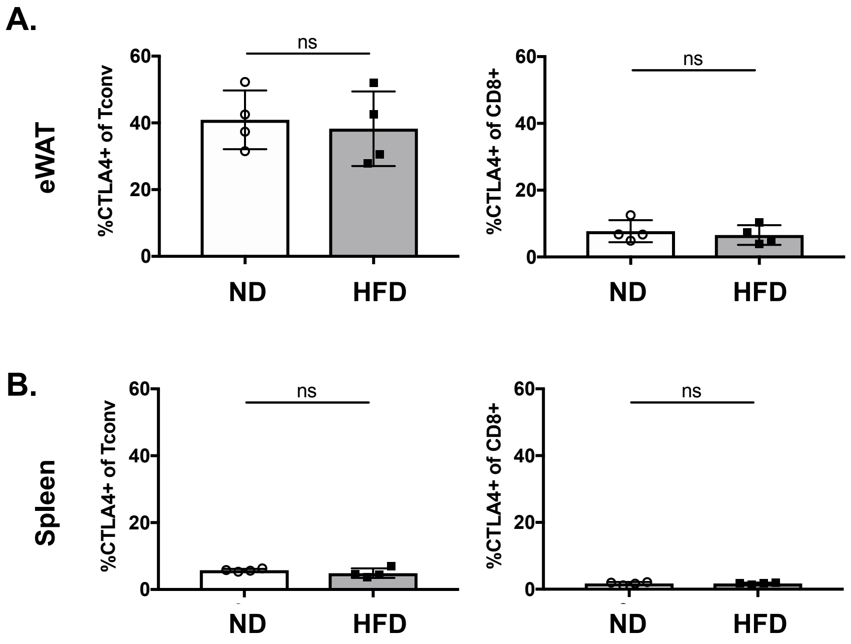

Supplemental Figure 4: CTLA-4 and PDL1 expression on eWAT and splenocytes.

(**A**) Frequency of CTLA-4 expression on freshly isolated Tconv and CD8+ eWAT ATTs and,

(**B**) spleen, measured by flow cytometry.

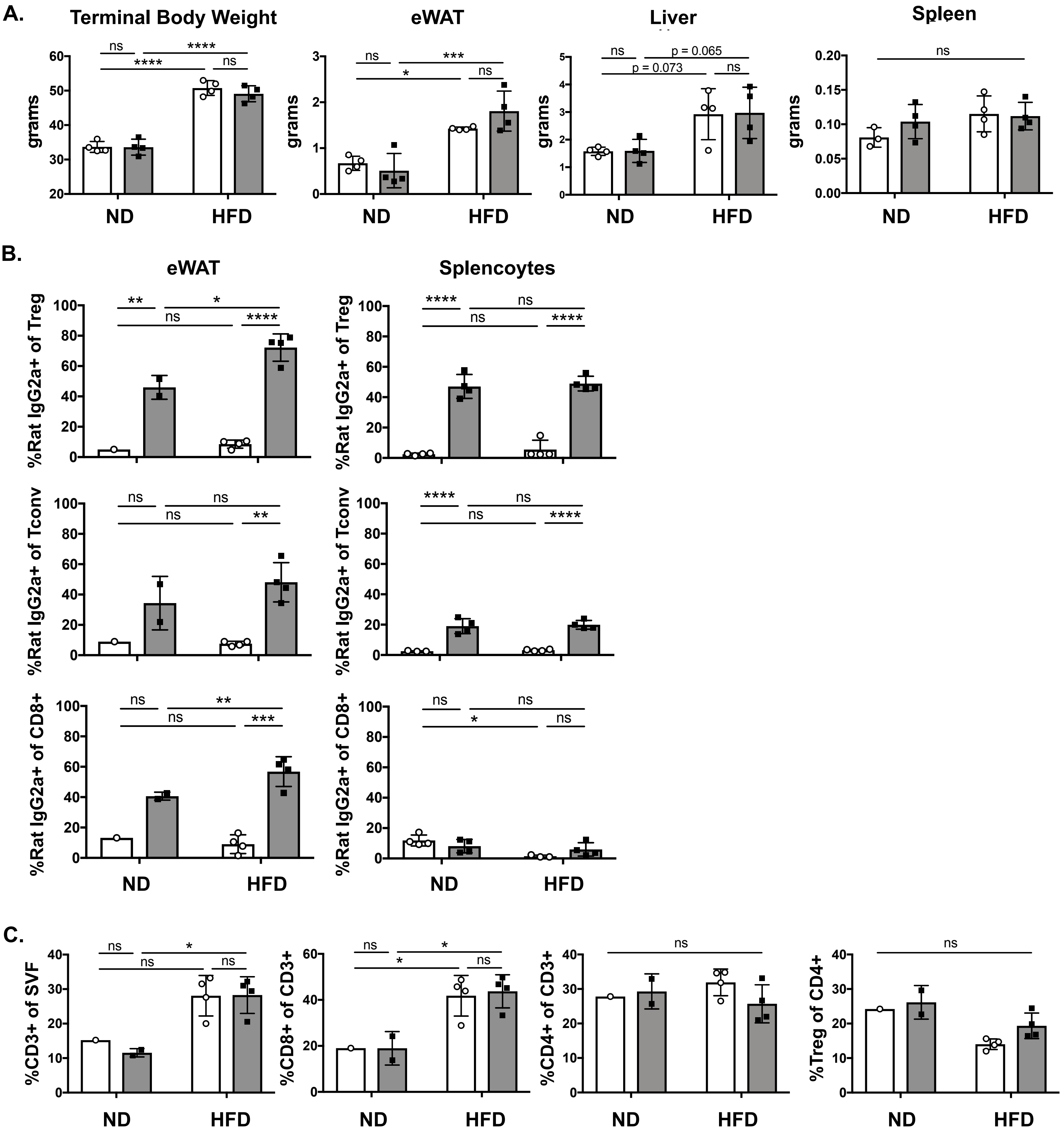

Supplemental Figure 5: PD1 blockade phenotyping data of mice fed HFD for 18 weeks.

(**A**) Terminal body and organ weights of PD1 blockade mice after IgG2a or PD1 antibody injections.

(**B**) Flow cytometric analysis of fresh non-cultured ATT cells after PD1 blockade.

(**C**) Flow cytometric measurement of T cells bound by Rat IgG2a (in-vivo PD1 antibody).

* = $p < 0.05$, ** = $p < 0.01$, *** = $p < 0.001$, **** = $p < 0.0001$

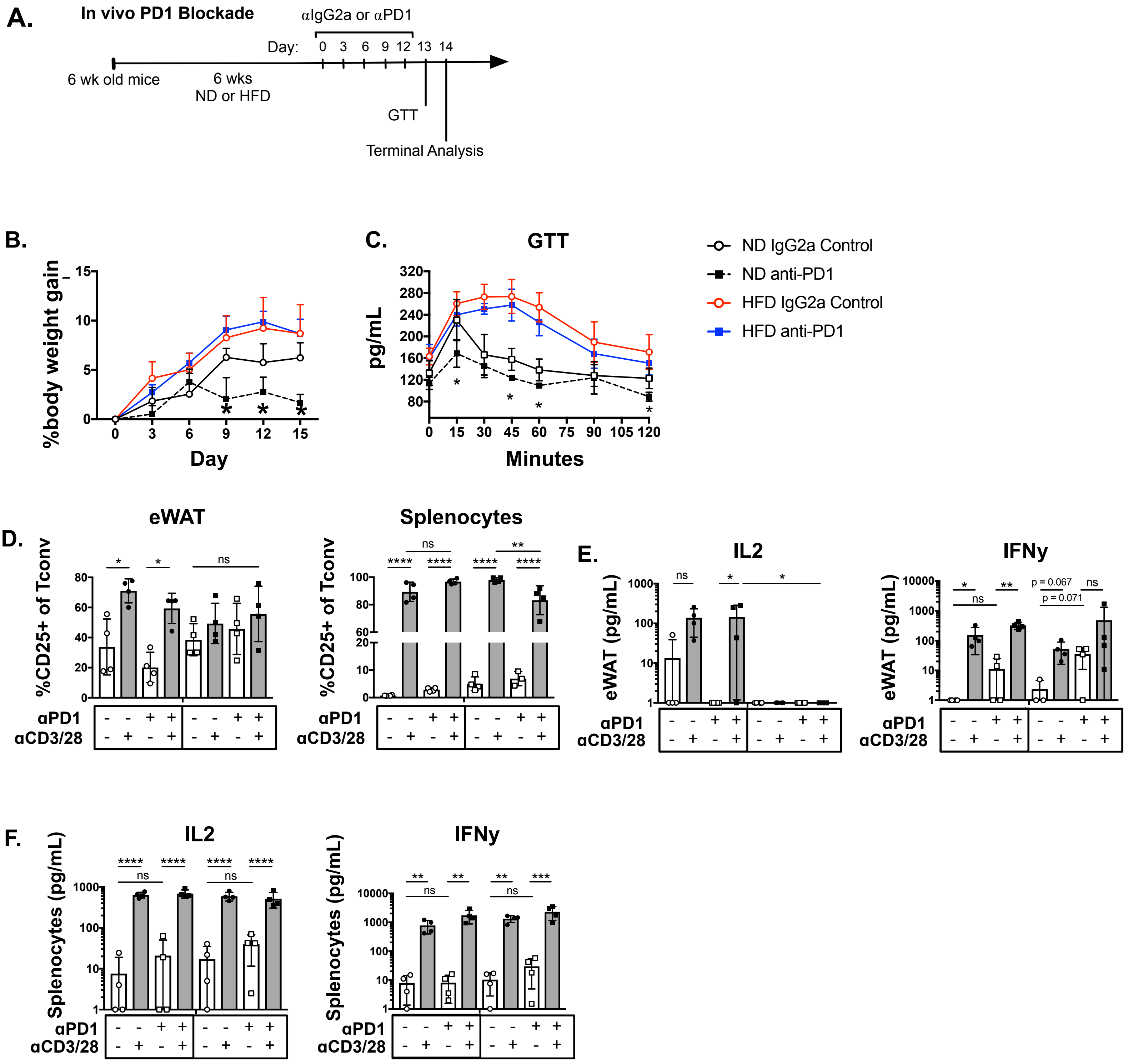

Supplemental Figure 6: PD1 blockade does not reverse ATT impairment after 6 weeks of HFD feeding.

(**A**) Model of PD1 blockade experiment. (**B**) Body weight changes of ND or HFD mice during the time course of IgG2a or PD1 antibody injections. (**C**) GTT after completed the day after a regimen of PD1 blockade. (**D**) CD25 expression on Tconv cells from eWAT and SPL after ATT activation assay. (**E**) IL2 and IFNy content in eWAT supernatants and, (**F**) splenocyte supernatants after ATT activation assays.

* = $p < 0.05$, ** = $p < 0.01$, *** = $p < 0.001$, **** = $p < 0.0001$

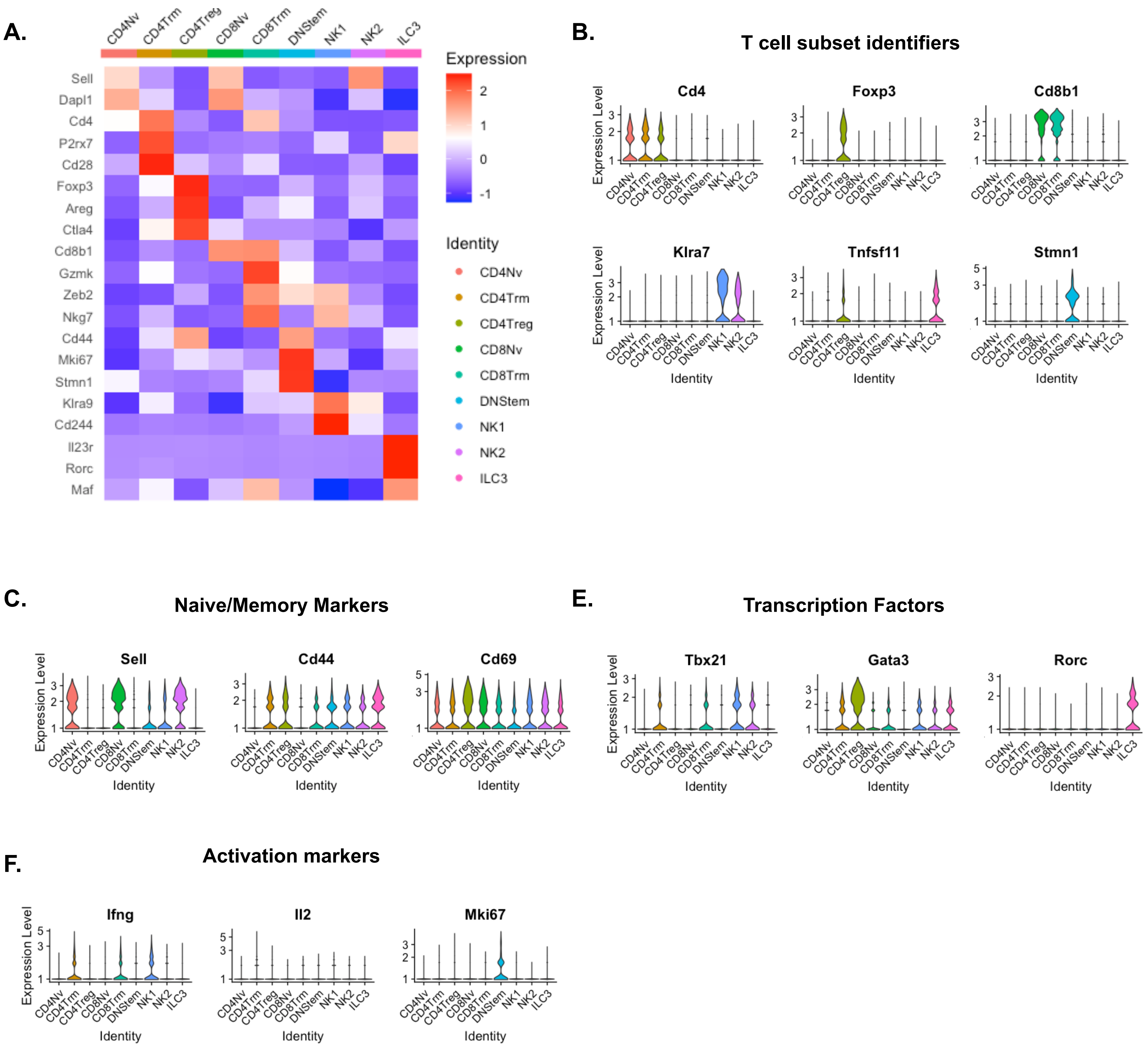

Supplemental Figure 7: SC RNAseq of CD45+ CD3+ cells reveals 9 distinct ATT subsets
(**A**) Each cluster was compared and enriched genes for each group were used to determine the identity of each group. This heatmap shows gene of interest used to determine each cluster. (**B**) Violin plots showing gene expression markers associated with T cell and ILCs (**C**) Violin plots of naïve and memory T cell markers. (**D**) Violin plots of transcription factors used to distinguish Tconv and ILC subsets. (**E**) Violin plots of effector cytokines and markers of proliferation used to classify T cell activation and inflammation of ATT subsets.
